# Dietary caffeine to assess CYP1A2 activity, tailor clozapine doses, and predict treatment response: genetic, epigenetic and clinical analyses

**DOI:** 10.1038/s41380-025-03256-x

**Published:** 2025-09-17

**Authors:** Nermine Laaboub, Frederik Vandenberghe, Nicolas Ansermot, Marianna Piras, Setareh Ranjbar, Dusan Petrovic, Giorgio Pistis, Sophie Vandenberghe-Dürr, Marie-Pierre F. Strippoli, Pedro Marques-Vidal, Belen Ponte, Menno Pruijm, Bruno Vogt, Franziska Gamma, Armin von Gunten, Kerstin Jessica Plessen, Philippe Conus, Séverine Crettol, Peter Vollenweider, Martin Preisig, Murielle Bochud, Chin B. Eap

**Affiliations:** 1 Unit of Pharmacogenetics and Clinical Psychopharmacology, Department of Psychiatry, Centre for Psychiatric Neuroscience, Lausanne University Hospital, University of Lausanne, Prilly, Switzerland; 2 Psychiatric Epidemiology and Psychopathology Research Center, Department of Psychiatry, Lausanne University Hospital and University of Lausanne, Prilly, Switzerland; 3 Centre for Primary Care and Public Health (Unisanté), University of Lausanne, Lausanne, Switzerland; 4 Division of Immunology and Allergology, Department of Medicine, University Hospitals of Geneva, Geneva, Switzerland; 5 Department of Medicine, Internal Medicine, Lausanne University Hospital and University of Lausanne, Lausanne, Switzerland; 6 Division of Nephrology, Geneva University Hospitals, Geneva, Switzerland; 7 Service of Nephrology and Hypertension, University Hospital of Lausanne and University of Lausanne, Lausanne, Switzerland; 8 Department of Nephrology and Hypertension, Inselspital, Bern University Hospital, University of Bern, Bern, Switzerland; 9 Les Toises Psychiatry and Psychotherapy Center, Lausanne, Switzerland; 10 Service of Old Age Psychiatry, Department of Psychiatry, Lausanne University Hospital, University of Lausanne, Prilly, Switzerland; 11 Service of Child and Adolescent Psychiatry, Department of Psychiatry, Lausanne University Hospital, University of Lausanne, Prilly, Switzerland; 12 Service of General Psychiatry, Department of Psychiatry, Lausanne University Hospital, University of Lausanne, Prilly, Switzerland; 13 University of Lausanne, Lausanne, Switzerland

**Keywords:** Schizophrenia, Genetics, Biochemistry

## Abstract

Caffeine metabolic ratios (CMR) following monitored caffeine intake are the gold standard to probe cytochrome P450 (CYP) 1A2 activity, which metabolizes antipsychotics like clozapine and olanzapine. Given caffeine’s ubiquity, we tested whether random CMR from dietary caffeine were associated with (1) clinical, genetic, and epigenetic factors linked to CYP1A2 activity; (2) plasma concentrations of clozapine and olanzapine; and (3) psychotropic treatment response. First, we analyzed two population-based studies (CoLaus|PsyCoLaus, N = 4898; SKIPOGH, N = 2054) to investigate random CMR associations with clinical, genome-wide, and epigenome-wide factors associated with CYP1A2 activity. Second, in psychiatric cohorts, we tested CMR associations with dose-normalized plasma concentrations (C/D) of clozapine (N = 164) and olanzapine (N = 222) and with psychotropic treatment response, including hospital admission risk (N = 1019) and prolonged stays (N = 1349). CMR were positively associated with age, CYP1A2 inducers including smoking, and negatively with female sex. CMR were negatively associated with clozapine C/D, explaining up to 14.9% of the variance; over six-fold the variance explained by genetic factors. A one-unit increase in CMR was associated with a 26% increased likelihood of hospital admission (p = 0.002) and reduced short-stay chance by 11% (p < 10^−3^). Random CMR provides a useful method to probe CYP1A2 activity, contributing, alongside other variables, to personalizing clozapine doses and identifying psychiatric patients at risk of hospital admission and lengthy stays. Incorporating routine measurement of random CMR before introduction of clozapine could be considered to allow early assessment of CYP1A2 activity, a key determinant of personalized clozapine dose titration.

## Introduction

Cytochrome P450 1A2 (CYP1A2) is responsible for 10–15% of drug metabolism [1], including antipsychotics (e.g., clozapine, olanzapine), antidepressants (e.g., duloxetine), and xenobiotics (e.g., caffeine). CYP1A2 activity is influenced by several factors, namely smoking, sex, advanced age, inflammation, drug interaction (e.g., inhibition by fluvoxamine), and polymorphisms located in the *CYP1A2* gene (e.g., rs2472297) or other genes/regulatory regions (e.g., Aryl-Hydrocarbon receptor (*AHR*) gene, namely rs4410790) [1–4]. In addition, epigenetics, specifically DNA methylation, regulate *CYP1A2* gene expression [5]. Importantly, variation in CYP1A2 activity can increase the risk of non-response to psychotropic treatment or toxicity, which could lead to hospital admission and prolonged lengths of stay (LOS). Thus, assessing CYP1A2 activity when prescribing its substrates is therefore highly relevant for optimizing treatment [1]. Genotyping and phenotyping are the two methods used to assess the in-vivo activity of drug-metabolizing enzymes. By integrating the effects of clinical, environmental, and genetic factors, phenotyping with an enzyme-specific compound can provide a more accurate estimation of enzyme activity than genotyping [6]. Considering that CYP1A2 is responsible for over 95% of caffeine metabolism to paraxanthine, the determination of plasma paraxanthine-to-caffeine ratios (caffeine metabolic ratios or CMR) is widely used to probe CYP1A2 activity [7]. In practice, after 12–48 h abstinence from caffeine, phenotyping is performed 2–7 h after the intake of 50–200 mg of caffeine [2, 7]. However, intentionally or not, not all participants comply with this procedure. Thus, individuals who self-reported abstinence from caffeine might exhibit elevated plasma levels of caffeine and its metabolites [8]. Because of the ubiquitous dietary consumption of caffeine and the use of a metabolic ratio instead of an absolute concentration of caffeine, the determination of CMR in plasma samples drawn at random times, without specific administration of caffeine, may therefore offer a more convenient approach for studies estimating CYP1A2 activity.

Furthermore, clozapine and olanzapine are among the most effective antipsychotics used to treat schizophrenia [9]. These drugs are mainly metabolized by CYP1A2, with contributions of CYP3A4 and CYP2C19 for clozapine to form its main metabolite, norclozapine, and a major contribution from uridine diphosphate glucuronosyltransferase (UGT) 1A4 for olanzapine [10]. Therapeutic reference range for plasma levels are established for clozapine (350–600 ng/mL), with an upper limit being related to an increased risk of tonic-clonic seizures [10]. The suggested reference range for olanzapine is 20–80 ng/mL [10]. A new range of 20–40 ng/mL has been recently proposed based on a systematic review of the available data [11]. Because of their high pharmacokinetic variabilities, explained in part by the variation of CYP1A2 activity, and because of their serious side-effects [9], clozapine and olanzapine are good candidates for dose personalization. Therapeutic drug monitoring (TDM) of these antipsychotics is thus strongly recommended [10], while dose adjustments by preemptive genotyping of *CYP1A2* and/or of other genes are not, because of the low pharmacokinetic variance explained by genetic variants [12, 13]. Interestingly, a recent genome-wide association study (GWAS) on clozapine levels found several genetic variants to be associated with clozapine and norclozapine blood levels, and clozapine-to-norclozapine metabolic ratios [14]. However, polygenic risk scores explained only 0.61, 1.6, and 7.3% of the variance, respectively, probably precluding their clinical usefulness for the personalization of clozapine doses.

Therefore, since monitored CMR is clinically used to probe CYP1A2 activity and considering the ubiquitous presence of caffeine, the first objective of this study is to investigate the associations of the well-known clinical and genetic factors explaining the variation of monitored CMR with random CMR in two population-based samples (SKIPOGH and CoLaus|PsyCoLaus). In addition, GWAS and epigenome-wide association study (EWAS) analyses were performed to determine genetic and epigenetic variants associated with CMR. The second objective was to test associations of CMR with genetic variants and with clozapine and olanzapine plasma levels, to explore possible clinical relevance of random CMR for dosage personalization in two psychiatric cohorts. Third, considering the potential impact of variations of drug plasma levels on the risk of relapse, the associations between CMR and response to psychotropic treatment were assessed by the likelihood of psychiatric admission and prolonged LOS.

## Methods

### Study designs, population, and settings

Data from four Swiss cohorts were analyzed in this study (detailed methodology provided in Supplementary Methods).

- SKIPOGH (Swiss Kidney Project on Genes in Hypertension) [15]: A family- and population-based study investigating genetic and environmental influences on blood pressure. The study was performed in two waves (baseline 2009–2013, N = 1123; 3-year follow-up 2012–2016, N = 931) that were merged together for analysis. Ethical approval was obtained from the ethics committees of Lausanne (92/07 and 303/12), Geneva (09-089 and 12-286), and Bern (091/09 and 015/13) for the baseline and second waves, respectively.
- CoLaus | PsyCoLaus [16]: A population-based cohort from Lausanne focused on mental disorders and cardiometabolic risk factors (Approval number CER VD = 16/03). From the 5064 adults enrolled between 2009 and 2013, we analyzed data from 4898 participants with available caffeine and paraxanthine measurements.
- PsyMetab [17]: A longitudinal study collecting clinical and genetic data from psychiatric patients receiving weight-gain-inducing psychotropic medications (Approval number CER VD = 2017-01301). Participants were recruited between 2007 and 2023 from both inpatient and outpatient services at Lausanne University Hospital and the Centre “Les Toises”. Patient selection criteria for the clozapine, olanzapine, psychiatric admission, and prolonged length of stay (LOS) samples are provided in Supplementary Methods.
- Clozapine Pharmacokinetic Study [18]: Conducted in 2003 at clinical sites in Königsfelden and Lausanne, this study included 52 of 75 inpatients who met the inclusion criteria (e.g., absence of medications known to interact with clozapine metabolism; approved by the ethical committees of Königsfelden and Prilly-Lausanne in 2008).

All participants (or their legal representatives) provided written informed consent prior to enrollment. Each study protocol received approval from the relevant cantonal ethics committees.

### Random CMR

In all studies, random CMR were calculated by dividing paraxanthine by caffeine plasma levels expressed in ng/mL and multiplying the ratio by 1.07769 (molecular mass ratio), without specific administration of caffeine and thereby reflecting people’s usual dietary intake. Using blood samples drawn after an overnight fast from the first follow-up of CoLaus|PsyCoLaus study, both waves of SKIPOGH, PsyMetab follow-up, and from the clozapine pharmacokinetic study, plasma levels of caffeine and paraxanthine were quantified by ultra-high performance liquid chromatography (Waters ACQUITY system) coupled to a tandem quadrupole mass spectrometer (Waters TQD with electrospray ionization or Waters Xevo TQ-S with UniSpray ion source). The method was validated according to international guidelines using a stable isotope-labeled internal standard for each analyte with a limit of quantification of 5 ng/mL (detailed method available on request). Of note, caffeine and paraxanthine were quantified in the same samples as clozapine or olanzapine quantification for PsyMetab and clozapine pharmacokinetic studies.

### Factors associated with CMR

#### Clinical factors

Covariates associated with paraxanthine and caffeine levels or with CYP1A2 activity, based on a priori knowledge, were identified, and adjusted for in the multivariable models (Supplementary Table 1). The time interval between blood drawing and last caffeine intake was only available in SKIPOGH and was considered in analyses. For the clozapine and olanzapine analysis, age, sex, body mass index (BMI), high-sensitivity C-reactive protein (hsCRP), and smoking status were included as covariates, while analyses for hospital admission and LOS were adjusted for age, sex, smoking, and psychiatric diagnoses.

#### Genetic variation of CYP1A2 activity

First, in CoLaus|PsyCoLaus and SKIPOGH, analyses were adjusted for 33 single nucleotide polymorphisms (SNPs) potentially associated with CYP1A2 activity and showing less than 80% linkage disequilibrium as covariates for CMR (see Supplementary Table 2 for the SNPs list and references).

Subsequently, GWAS were performed in CoLaus|PsyCoLaus (N = 3762) to identify genetic variants associated with CMR (see Supplementary Methods for detailed descriptions of the analyses).

Finally, SNPs significantly associated with CMR in the CoLaus|PsyCoLaus GWAS were tested in the psychiatric cohorts for their association with clozapine dose-normalized plasma concentrations (C/D), norclozapine C/D, clozapine/norclozapine, olanzapine C/D, admission to hospital, and LOS.

#### Epigenetic variation of CYP1A2 activity (EWAS)

Epigenome-wide DNA methylation from white blood cells was measured in SKIPOGH2 participants (second study wave; N = 565) using two different microarrays (Supplementary Methods). In the present analyses, 452′453 CpG sites available across both arrays were used.

### Clozapine, norclozapine, and olanzapine plasma level determinations

For the PsyMetab study, plasma concentrations of clozapine, norclozapine, and olanzapine were quantified by high performance liquid chromatography coupled to mass spectrometry or ultra-high performance liquid chromatography coupled to tandem mass spectrometry (Supplementary Methods). For the clozapine pharmacokinetic study, a gas chromatography with a nitrogen-phosphorus detector method was used [18]. All observations were at steady state, with blood sampling performed between 5–24 and 9–26 h after last clozapine and olanzapine intake, respectively.

### Statistical analyses

#### Associations between CMR, clinical covariates, and SNPs

CMR were box-cox transformed to better approximate a normal distribution in SKIPOGH and CoLaus|PsyCoLaus, given their skewed distributions, and serve as outcome measures. Linear mixed-effect regression models with a nested random effect structure (participant observations nested within family; SKIPOGH) and linear regression models (CoLaus|PsyCoLaus) were applied to examine the association between CMR and clinical covariates selected by a backward selection procedure. Of note, due to the limited number of participants receiving strong CYP1A2 inhibitors (i.e., ciprofloxacin, fluvoxamine, and norfloxacin), 7 (0.3%) and 4 (0.1%) in SKIPOGH and CoLaus|PsyCoLaus, respectively, they were excluded from multivariable analyses. In all selection procedures for clinical relevance, age, sex, and CYP1A2 inducers were forced into the models. The final model formulas are detailed in the Supplementary File. Participants of Caucasian descent were selected to test the relationship between SNP candidates and CMR, considering the variables selected using the Bayesian Information Criterion. Finally, sensitivity analyses were performed using the same model in each cohort by excluding the 5^th^ or 95^th^ percentiles of CMR, caffeine, paraxanthine, and caffeine plus paraxanthine, respectively, and by analyzing the first and second SKIPOGH waves separately.

#### Associations between CMR, clozapine, norclozapine, and olanzapine plasma levels

Clozapine C/D, norclozapine C/D, clozapine/norclozapine, and olanzapine C/D were log transformed to better approximate a normal distribution. Linear mixed-effect regression models were applied to examine the association between clozapine C/D, norclozapine C/D, clozapine/norclozapine, and olanzapine C/D and CMR adjusted by clinical factors and SNPs previously associated with CMR. Receiver operating characteristic (ROC) curves were performed to identify a CMR threshold predicting at best a twofold increase in (reflecting a slower metabolism) and a halving of (reflecting a faster metabolism) clozapine or olanzapine C/D.

#### Associations between CMR, psychiatric admission risk, and LOS

Logistic regression models with fixed effects were used to test associations between CMR and the odds of experiencing at least one hospital admission over one year, while mixed-effect Cox regression models were used to test associations between LOS and CMR. Both analyses were performed adjusting for the aforementioned covariates and the significant SNPs identified in the CoLaus|PsyCoLaus GWAS.

Analyses were conducted using Stata 16.1 (StataCorp; College Station, TX, USA), and the R environment for statistical computing, version 4.1.1. *P* values ≤ 0.05 were accepted as statistically significant.

## Results

### Description of the cohorts

Demographic and clinical characteristics of SKIPOGH (N = 2054) and CoLaus|PsyCoLaus (N = 4898) are presented in Supplementary Table 3. CoLaus|PsyCoLaus participants were older, had higher BMI, and higher proportions were alcohol consumers, participants meeting hypertension criteria, and statin users than those in SKIPOGH (all p < 10^−3^). In contrast, SKIPOGH participants had higher eGFR and included more current smokers (both p ≤ 0.006). Concerning CYP1A2 activity, median CMR and the proportion of subjects with CYP1A2 inducers were similar in both studies (p = 0.91), while the proportion of subjects with CYP1A2 inhibitors was higher in CoLaus|PsyCoLaus (p = 0.001). Of note, only seven (0.3%) of SKIPOGH and 39 (0.8%) of CoLaus|PsyCoLaus participants had undetectable plasma levels of caffeine and/or paraxanthine, respectively (46 patients excluded). CMR did not differ between Caucasians and non-Caucasians (p_SKIPOGH_ = 0.29; p_CoLaus|PsyCoLaus_ = 0.25).

Demographic and clinical characteristics of clozapine (PsyMetab and clozapine pharmacokinetic study) and olanzapine (PsyMetab) samples are given in Table 1. Patients were treated with a median (range) daily dose of 300 (13-900) mg and 15 (3-35) mg of clozapine and olanzapine, respectively. Median (range) plasma concentrations were 282 (8-1140) ng/mL and 27 (1-138) ng/mL for clozapine and olanzapine, respectively, with a similar median CMR of 1.1 and an overall range of 0.1–6.2. Except for daily doses, drug dosage regimen, time between drug intake and blood sampling, and clozapine/norclozapine plasma concentration ratios, no significant differences were observed between the clozapine pharmacokinetic study and the PsyMetab study (Supplementary Table 4). Caucasians had significantly lower CMR than non-Caucasians in both clozapine (p = 0.04) and olanzapine (p = 0.03) PsyMetab cohorts (see Supplementary Tables 5 and 6).

**Table 1:** Demographic and clinical characteristics of clozapine (PsyMetab and clozapine pharmacokinetic study) and olanzapine (PsyMetab) samples.

Baseline characteristics	Clozapine	Olanzapine
	N = 120	N = 146
**Age** (years; median [IQR])	49 [37, 67]	39 [28, 54]
Range (min-max)	(17–90)	(13–90)
**Sex** (N (%))		
Male	62 (52%)	77 (53%)
**Current smokers** (N (%))	55 (46%)	77 (53%)
**BMI** (kg/m^2^; median [IQR])	25.4 [23.1, 29.1]	24.0 [21.3, 27.4]
Range (min-max)	(14.2–37.5)	(13.7–42.9)

Observational characteristics	N = 164	N = 222
**Daily dose** (mg/day; median [IQR])	300 [150, 400]	15 [10, 20]
Range (min-max)	(13–900)	(3–35)
**Drug dosage regimen**		
1 time daily	59 (36%)	
2 times daily	74 (45%)	
3 times daily	19 (12%)	
4 times daily	12 (7%)	
**Time between drug intake and blood sampling** (hours; median [IQR])	13.1 [11.0, 14.0]	13.5 [12.0, 14.5]
Range (min-max)	(5.5–24.0)	(9.0–26.0)
**Drug plasma concentration** (ng/mL; median [IQR])	282 [139, 460]	27 [14, 42]
Range (min-max)	(8–1140)	(1–138)
**Drug plasma concentration / dose ratios** ((ng/ml)/mg; median [IQR])	1.1 [0.7, 1.5]	2.1 [1.5, 2.9]
Range (min-max)	(0.1–5.4)	(0.1–6.4)
**Norclozapine plasma concentration** (ng/mL; median [IQR])	148 [67, 260]	
Range (min-max)	(7–607)	
**Clozapine / norclozapine plasma concentration ratios** (median [IQR])	1.71 [1.41, 2.23]	
Range (min-max)	(0.72–8.47)	
**Caffeine metabolic ratios**^a^ (median [IQR])	1.1 [0.8, 1.9]	1.1 [0.7, 2.1]
Range (min-max)	(0.2–4.5)	(0.1–6.2)
**High sensitivity C-reactive protein** (mg/L; median [IQR])	2.3 [0.9, 5.1]	2.2 [0.9, 5.2]
Range (min-max)	(0.2–136.4)	(0.1–50.3)
Unknown	52	87

*BMI* body mass index, *IQR* interquartile range, *kg* kilograms, *L* liter, *m*^*2*^ square meter, *mg* milligram, *min* minimum, *max* maximum, *mL* milliliter, *N* number, *ng* nanogram.

^a^Calculated by dividing paraxanthine by caffeine plasma levels expressed in ng/ml and multiplying the ratio by 1.07769 (Molecular mass ratio).

Demographic and clinical characteristics of PsyMetab patients included in hospital admission and LOS analyses are given in Supplementary Table 7. The cohort primarily consisted of patients diagnosed with mood and psychotic disorders, and 65% of the patients had been admitted to the hospital at least once, with a median duration of stay of 42 days.

### Association between CMR, clinical covariates, and SNPs in SKIPOGH and CoLaus|PsyCoLaus studies

**Fig. 1:**
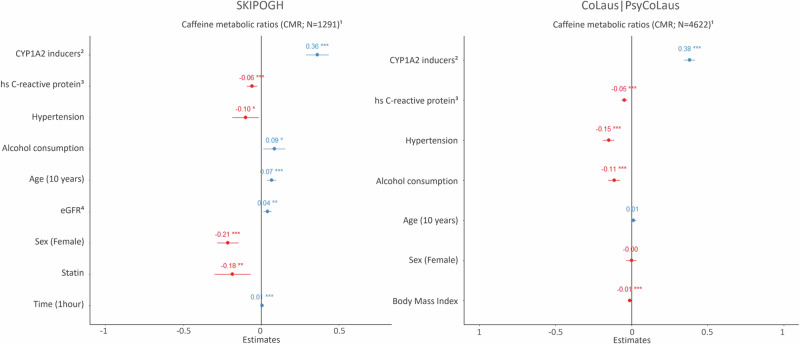
Associations between caffeine metabolic ratios, clinical covariates in SKIPOGH and CoLaus|PsyCoLaus. Number of observations varies due to missing values. Covariates selected using backward procedure. Of note, due to the design of the SKIPOGH study (i.e., a multicenter study), the center effect was included in the analyses; however, as it failed to show statistically significant effect, it was excluded from the model. *p < 0.05, ** p < 0.01, *** p < 0.001. ^1^ Box-cox transformed to insure normal distribution. ^2^ Including smoking. ^3^ log transformed to better approximate a normal distribution. ^4^ The kidney function was estimated by the glomerular filtration rate (eGFR) calculated using Chronic Kidney Disease - Epidemiology Collaboration formula (by 10 ml/min/1.73 m^2^). hs high sensitivity. Example of interpretations: In SKIPOGH, CYP1A2 inducer users and alcohol consumers, had a 36 and 9% increased CMR as compared to non-CYP1A2 activity inducer users and non-alcohol consumers, respectively. For each one unit increase in log hs C-reactive protein levels, CMR decreased by 6%, while for each 10-year increase in age and 10 ml/min/1.73 m^2^ increase in eGFR, CMR increased by 7 and 4%, respectively.

With respect to clinical factors associated with CYP1A2 activity (Fig. 1), both cohorts showed positive associations of CMR with CYP1A2 inducers (β_SKIPOGH_ = 0.36, 95% CI_SKIPOGH_ = [0.29, 0.44]; β_Colaus|PsyCoLaus_ = 0.38, 95% CI_Colaus|PsyCoLaus_ = [0.35, 0.42]), as well as negative associations of CMR with hsCRP (β_SKIPOGH_ = −0.06, 95% CI_SKIPOGH_ = [−0.09, −0.02]; β_Colaus|PsyCoLaus_ = −0.05, 95% CI_Colaus|PsyCoLaus_ = [−0.07, −0.03]) and with arterial hypertension (β_SKIPOGH_ = −0.10, 95% CI_SKIPOGH_ = [−0.18, −0.02]; β_Colaus|PsyCoLaus_ = −0.15, 95% CI_Colaus|PsyCoLaus_ = [−0.19, −0.11]). The relationship between CMR and alcohol consumption was discordant in the two cohorts (β_SKIPOGH_ = 0.09, 95% CI_SKIPOGH_ = [0.01, 0.16]; β_Colaus|PsyCoLaus_ = −0.11, 95% CI_Colaus|PsyCoLaus_ = [−0.15, −0.08]). In CoLaus|PsyCoLaus, elevated BMI was negatively associated with CMR (β_Colaus|PsyCoLaus_ = −0.01, 95% CI_Colaus|PsyCoLaus_ = [−0.02, −0.01]), while no significant association was found in SKIPOGH (variable was not included in the final model as a result of the backward selection process). In SKIPOGH, increased age (β = 0.07, 95% CI = [0.04, 0.10], for each 10 years increase) and elevated eGFR (β = 0.04, 95% CI = [0.01, 0.07], for each 10 ml/min/1,73 m^2^ increase) were positively associated with CMR, while female sex (β = −0.21, 95% CI = [−0.28, −0.14]) and statin use (β = −0.18, 95% CI = [−0.30, −0.07]) were both negatively related to CMR. Finally, a positive association was observed between the time from last caffeine intake to blood sampling and CMR (β = 0.01, 95% CI = [0.00, 0.01], for each additional hour; data available only in SKIPOGH).

Considering the genetic variation of *CYP1A2* (analyzed SNPs) in both cohorts, rs2472297 T allele and rs4410790 T allele were positively and negatively associated with CMR, respectively (Supplementary Figs. 1, 2).

Finally, the results remained consistent in sensitivity analyses when analyzing the SKIPOGH waves separately, or when excluding in SKIPOGH and CoLaus|PsyCoLaus the 5th or 95th percentile of only caffeine or paraxanthine or both caffeine and paraxanthine, whether the variables associated with CMR included or excluded SNPs (data not shown).

### Genome-wide association analysis (GWAS)

**Fig. 2:**
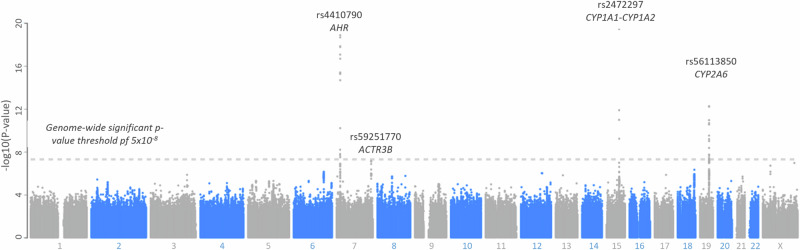
GWAS-significant SNPs associated with caffeine metabolic ratios in CoLaus|PsyCoLaus (N = 3762).

GWAS analyses also identified the two SNPs associated with CMR: rs2472297, rs4410790, and rs56113850, with the C allele of rs56113850 showing a negative association with CMR (nearest gene *CYP2A6*). Furthermore, rs59251770 (nearest gene *ACTR3B*) was identified at a borderline-significant level (Fig. 2 and Supplementary Table 8).

### Epigenome-wide association analysis (EWAS)

EWAS analyses identified two CpGs associated with CMR at the Benjamini-Hochberg threshold, namely cg23684074 located within *FLNC* gene on chromosome 7, and the intergenic cg05817750 on chromosome 17, both CpGs being demethylated in relation to the phenotype of interest (β < 0; Supplementary Fig. 3).

### Associations between CMR with clozapine and olanzapine C/D ratios

Table 2 shows associations of CMR with clozapine C/D, norclozapine C/D, clozapine/norclozapine, and olanzapine C/D ratios. For one unit of CMR increase, log clozapine C/D decreased by 0.30 (95% CI = [−0.40, −0.20]), corresponding to an approximate 26% decreases in the actual concentration ratios (95% CI = [−18%, −33%]); log norclozapine C/D decreased by 0.21 (95% CI = [−0.31, −0.11]) or a 19% decrease (95% CI = [−10%, −27%]); and log clozapine/norclozapine decreased by 0.09 (95% CI = [−0.16, −0.02]), or a 9% decrease (95% CI = [−2%, −15%]). No significant association was found between CMR and log olanzapine C/D ratios (β = −0.04; 95% CI = [−0.10, 0.02]). CMR explained 14.9% of clozapine C/D variability, but only 0.5% of olanzapine C/D variability. An online application is available to visualize the effect of CMR and other parameters on clozapine and olanzapine C/D ratios (https://uppc.shinyapps.io/diet_cmr/). In exploring factors associated with log clozapine C/D, we also examined log clozapine-to-norclozapine ratios, commonly cited as proxies for CYP1A2 enzymatic activity. These ratios showed a significant association with log clozapine C/D (β = 0.14, 95% CI = [0.04, 0.23]), but with a lower explained variability (4.2%) as compared to CMR (14.9%; Supplementary Table 9).

**Table 2:** Associations between caffeine metabolite ratios with clozapine, norclozapine, and olanzapine plasma concentration-to-dose ratios and with clozapine-to-norclozapine ratios.

	Clozapine [ng/mL] / dose [mg/d]^a^			Norclozapine [ng/mL] / dose [mg/d]^a^			Clozapine [ng/mL] / Norclozapine [ng/mL]^a^			Olanzapine [ng/mL] / dose [mg/d]^a^		
*Predictors*	*Estimates*	*95% CI*	*p-value*	*Estimates*	*95% CI*	*p-value*	*Estimates*	*95% CI*	*p-value*	*Estimates*	*95% CI*	*p-value*
(Intercept)	−0.23	−0.97 – 0.52	0.55	−0.57	−1.37 − 0.23	0.16	0.30	−0.26 – 0.86	0.29	0.47	−0.07 – 1.02	0.09
Caffeine metabolic ratios^b^	−0.30	−0.40 – −0.20	**<0.001**	−0.21	−0.31 – −0.11	**<0.001**	−0.09	−0.16 – −0.02	**0.01**	−0.04	−0.10 – 0.02	0.21
Age (10 years)	0.08	0.03 – 0.13	**0.001**	0.09	0.03 – 0.14	**0.002**	−0.00	−0.04 – 0.03	0.84	0.05	−0.00 – 0.10	0.06
Sex (Female)	0.13	−0.05 – 0.31	0.17	0.11	−0.08 – 0.31	0.25	0.00	−0.14 – 0.14	0.98	0.27	0.10 – 0.44	**0.002**
Body Mass Index	0.03	0.01 – 0.05	**0.003**	0.02	−0.00 – 0.04	0.10	0.01	−0.00 – 0.03	0.10	0.02	0.00 – 0.03	**0.03**
Smoking	−0.24	−0.45 – −0.04	**0.02**	−0.37	−0.59 – −0.15	**0.001**	0.16	0.01 – 0.32	**0.04**	−0.41	−0.57 – −0.25	**<0.001**
Time (hours)^c^	−0.04	−0.07 – −0.01	**0.008**	−0.04	−0.07 – −0.01	**0.01**	0.00	−0.02 – 0.02	0.88	−0.02	−0.04 – 0.00	**0.04**
Patients	120			120			120			146		
Observations	164			164			164			222		
Marginal R^2^ / Conditional R^2^	0.399/0.577			0.339/0.616			0.093/0.577			0.247/0.749		
Partial R^2^ for:												
Caffeine metabolic ratios	14.94%			7.37%			3.17%			0.51%		
Age	5.36%			5.66%			0.05%			1.74%		
Sex	1.65%			1.36%			0.05%			3.67%		
Body Mass Index	5.04%			1.62%			2.21%			1.32%		
Smoking	2.83%			5.68%			2.72%			9.40%		
Time	2.59%			2.02%			0.00%			0.59%		

^a^Log transformed to better approximate a normal distribution.

^b^Calculated by dividing paraxanthine by caffeine plasma levels and multiplying the ratio by 1.07769 (Molecular mass ratio).

^c^Time spent between last clozapine or olanzapine intake and blood sampling in hours.

Significant *p*-values in bold. Of note, regression coefficients for log-transformed variables represent proportional changes. Percent change = (e^^β^−1) × 100. For example: β = −0.30 → (e^−0.30−1) × 100 = −25.9% (a 25.9% decrease).

*CI* Confidence Interval, *d* day, *mg* milligram, *ml* milliliter, *ng* nanogram.

The association between log clozapine C/D and CMR remained similar after adjusting for hsCRP (Supplementary Table 10). Additionally, to evaluate whether the drug dosage regimen (available for clozapine) influenced the results, analyses were conducted including this variable as a covariate. These analyses revealed no significant associations between the dosage regimen and clozapine C/D, norclozapine C/D, clozapine/norclozapine ratios. Importantly, including this covariate did not change the direction nor statistical significance of the main findings (data not shown). Similarly, a model including log clozapine trough concentrations, estimated from a mean half-life of 12 h and reported dosing regimen, showed no meaningful difference from the primary results (data not shown).

**Fig. 3:**
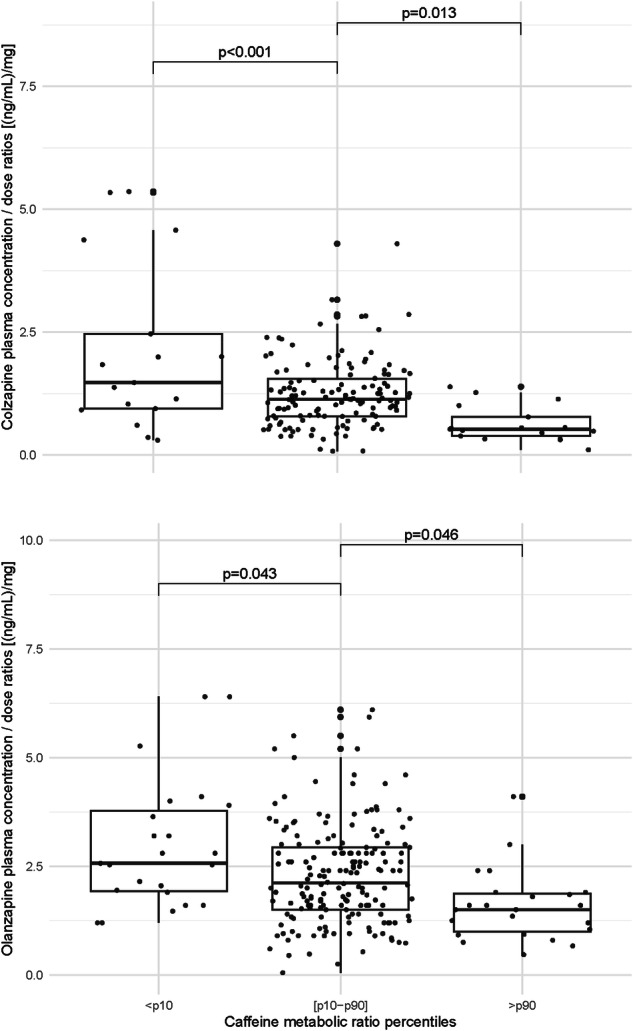
Clozapine and olanzapine concentration/dose ratios in the <10^th^ (slow metabolism), [10-90^th^] (normal metabolism) and >90^th^ percentiles (fast metabolism) of caffeine metabolic ratios. The *p*-values were obtained by fitting linear mixed-effects models. Boxplots display dose-adjusted plasma concentrations of clozapine (top) and olanzapine (bottom) stratified by caffeine metabolic ratio percentiles (< p10, [p10–p90], >p90). **For Clozapine: • <p10** (n = 17): Median = 1.48 ng/mL/mg; IQR = 1.52 (Q1 = 0.95, Q3 = 2.47).**• [p10–p90]** (n = 130): Median = 1.13 ng/mL/mg; IQR = 0.76 (Q1 = 0.79, Q3 = 1.6).**• >p90** (n = 17): Median = 0.53 ng/mL/mg; IQR = 0.39 (Q1 = 0.39, Q3 = 0.78). **For Olanzapine: • <p10** (n = 23): Median = 2.57 ng/mL/mg; IQR = 1.85 (Q1 = 1.92, Q3 = 3.77).**• [p10–p90]** (n = 176): Median = 2.12 ng/mL/mg; IQR = 1.43 (Q1 = 1.5, Q3 = 2.93).**• >p90** (n = 23): Median = 1.5 ng/mL/mg; IQR = 0.88 (Q1 = 0.99, Q3 = 1.88).

Patients with CMR lower than percentile 10 (CMR < 0.53 for clozapine and <0.43 for olanzapine, reflecting slow metabolism), or higher than percentile 90 (CMR > 2.53 for clozapine and >2.93 for olanzapine, reflecting fast metabolism), had clozapine and olanzapine C/D ratios significantly higher, and lower, respectively, than patients with CMR between percentiles 10 and 90 (normal metabolizers; Fig. 3).

Using ROC curves, CMR lower than 0.9, and lower than 0.6, discriminate clozapine and olanzapine C/D above twice the median value, respectively (slower metabolizers; AUC, specificity, and sensitivity: 0.78, 0.69, and 0.80 for clozapine; 0.71, 0.81, and 0.57 for olanzapine; Supplementary Fig. 4). CMR above 1.4 discriminate clozapine C/D below half the median value (faster metabolizers; AUC, specificity, and sensitivity: 0.80, 0.74, and 0.79). No CMR threshold discriminating olanzapine C/D below half the median value could be considered, as the obtained AUC was 0.56.

None of the previous identified SNPs associated with CMR (rs4410790, rs2472297, rs56113850) showed significant associations with clozapine C/D, norclozapine C/D, and olanzapine C/D ratios, either in Caucasians or across all ethnicities (Supplementary Tables 11 and 12). However, for rs2472297, a trend toward lower clozapine C/D was observed among patients carrying the C/T and T/T genotypes compared to CC carriers (β = −0.26, 95% CI = −0.52; 0.01, p = 0.06 in Caucasians; β = −0.24, 95% CI = −0.51; 0.03, p = 0.08 in all ethnicities). CMR and rs2472297 explained 5.7% and 0.0%, respectively, of clozapine C/D variability in Caucasians, and 13.0% and 2.1%, respectively, in all ethnicities. Of note, lower clozapine/norclozapine ratios were observed among patients carrying the rs2472297 C/T and T/T genotypes compared to CC carriers when considering all ethnicities (β = −0.17, 95% CI = −0.34; −0.00, p = 0.048; Supplementary Table 12). Additionally, a significant negative association between CMR and log olanzapine C/D ratios was found when adjusting for the aforementioned SNPs in Caucasians only (β = −0.11, 95% CI = [−0.19, −0.03]), corresponding to an approximate 10% decrease in olanzapine C/D ratios per unit increase in CMR (Supplementary Table 11).

### Associations between CMR, psychiatric admission, and LOS

Table 3 shows positive associations between CMR and psychiatric admission (N = 1019) and LOS (N = 1349). With each unit increase in CMR, the odds of experiencing at least one hospital admission increased by 26% (95% CI = [1.09, 1.46]), while the chance of experiencing a short LOS decreased by 11% (95% CI = [0.84, 0.95]). No CMR-related SNP was significantly associated with admission or LOS.

**Table 3:** Association between caffeine metabolic ratios, risk of at least one hospital admission and length of stays in PsyMetab.

	Hospital admission			Lengths of stay		
Predictors	Odds Ratios	95% CI	*p*-value	Hazard Ratios^c^	95% CI	*p*-value
(Intercept)	0.06	0.02–0.25	**<0.001**			
Age (10 years)	1.56	1.42–1.72	**<0.001**	0.87	0.84–0.90	**<0.001**
Sex (Male)	0.76	0.57–1.01	0.059	1.11	0.99–1.25	0.13
**Psychiatric diagnosis**^a^						
Mood disorders	1.54	1.06–2.26	**0.02**	1.00	0.83–1.21	0.99
Psychotic disorders	5.88	3.86–9.05	**<0.001**	0.87	0.72–1.06	0.28
Smoking	0.99	0.74–1.34	0.97	1.14	1.01–1.28	**0.05**
Caffeine metabolic ratios^b^	1.26	1.09–1.46	**0.002**	0.89	0.84–0.95	**<0.001**
rs2472297 (TT or CT; ref: CC)	0.85	0.61–1.19	0.35	0.98	0.86–1.12	0.79
rs4410790 (TT or CT; ref: CC)	1.34	0.99–1.82	0.057	0.99	0.88–1.11	0.88
rs56113850 (TT or CT; ref: CC)	1.21	0.36–4.01	0.75	0.82	0.44–1.54	0.38
Clozapine or olanzapine use	1.59	1.05–2.46	**0.03**	0.88	0.77–1.01	0.11
Patients	1019			664		
Observations	1019			1349		

Significant *p*-values in bold. Psychiatric diagnoses were defined as follow: psychotic disorders [F20-F25 and F28-F29], Mood disorders [F30-F31 and F32-F33], and other diagnoses [F00-F19, F34- F99].

^a^Compared to other diagnoses.

^b^Calculated by dividing paraxanthine by caffeine plasma levels and multiplying the ratio by 1.07769 (Molecular mass ratio).

^c^Hazard ratios > 1 indicated the higher likelihood of a short length of stay (i.e., length of stay below the median of length of stays of 42 days), whereas Hazard ratio < 1 indicated the risk of a prolonged length of stay (i.e., length of stay equal to or higher than the median of length of stays of 42 days).

*CI* Confidence Interval, *ref* reference.

## Discussion

### Main results

To our knowledge, the present study is the first to suggest that random CMR, with caffeine originating from diet without a monitored caffeine intake (i.e., standard phenotyping test), can be used to estimate CYP1A2 activity and might be considered alongside other variables to forecast and personalize doses of drugs predominantly metabolized by CYP1A2, namely clozapine, as well as to identify patients at risk of admission to a psychiatric hospital and prolonged LOS. This study is strengthened by the inclusion of over 5900 observations from two population-based studies, the analyses of several clinical, genetic, and epigenetic variant analyses, and the analysis of two psychiatric cohorts.

### Random caffeine use to estimate CYP1A2 activity

One previous study conducted in 19 healthy volunteers showed that CMR (composite ratios determined with several caffeine metabolites) from dietary caffeine, measured in random urine samples, are highly correlated with urinary CMR measured 6 h after a standardized intake of 100 mg caffeine [19]. In the present study, random CMR was found to be associated with clinical factors known to influence CYP1A2 activity. Smoking and the intake of drugs known to be CYP1A2 inducers were associated with higher CMR [1, 20]. No significant association was observed between CMR and drugs known to be CYP1A2 moderate inhibitors, which can probably be explained by the low prevalence of subjects with such inhibitors. In addition, the use of such drugs was self-reported, thus, memory bias cannot be ruled out, and the duration of drug exposure was not monitored. Elevated hsCRP levels were associated with lower CYP1A2 activity, which is in agreement with a recent study of 28 patients hospitalized with SARS-CoV-2 infection [2]. Hypertension was found to be inversely associated with CMR in both studies. These results must be interpreted with caution because of the multiple criteria used in the present study to define hypertension. However, one possible hypothesis is that the observed association is mediated by inflammation, which may contribute to hypertension [21], and reduces CYP1A2 activity. The effect of alcohol consumption on CYP1A2 activity was inconsistent between SKIPOGH (positive association) and CoLaus|PsyCoLaus (inverse association), which may be tentatively explained by the differences in age, sex distributions, and differences of alcohol-related questions between the two studies. In agreement with previous studies, female sex was associated with decreased CMR in SKIPOGH [20, 22]. The lack of such an association in CoLaus|PsyCoLaus may be due to the higher median age of women (57 years versus 49 years in SKIPOGH) and a higher proportion of women in menopause, CYP1A2 activity being decreased by female sex hormones [22]. High BMI was inversely associated with CMR in CoLaus|PsyCoLaus but not in SKIPOGH. A recent study (N = 30) found a positive association between BMI and CYP1A2 activity [2], whereas another study (N = 58) reported no significant association [23]. Of note, CoLaus|PsyCoLaus included participants with higher BMI and older age than SKIPOGH; future studies should investigate the effect of BMI on CMR, considering the pro-inflammatory state induced by obesity and increased age [24]. Importantly, CMR was found to be mostly independent of the time of caffeine intake. Thus, in SKIPOGH, although a positive association was found between CMR and time from last caffeine intake to blood sampling, accounting for this variable increased the explained variance by only 1.5%. Of note, blood samples were collected with respect to the fasting condition, implying a caffeine restriction for at least 8 h, which is significantly less than the recommended period of 12–48 h of caffeine restriction for phenotypic testing.

### Genetic variation of *CYP1A2* (analyzed SNPs and GWAS)

Concerning genetic factors, *CYP1A1-CYP1A2* rs2472297 T allele and *AHR* rs4410790 T allele were associated with increased and decreased CMR, respectively, which is in agreement with a study measuring olanzapine and its metabolite, desmethyl olanzapine [25]. Interestingly, GWAS analyses confirmed the association between CMR and the two abovementioned SNPs, and identified an additional genetic polymorphism associated with CMR, namely *CYP2A6* rs56113850. Rs56113850 has previously been associated with nicotine clearance as well as lower caffeine consumption [26, 27].

### Epigenetic variation of CYP1A2 (EWAS)

EWAS analysis showed that the FLNC- cg23684074 and cg05817750 markers are associated with CMR. Of note, no association was found with methylxanthine metabolism and FLNC- cg23684074 to date, while cg05817750 was previously related to kidney function and sex [28, 29], both related to CMR in SKIPOGH.

### Clozapine and olanzapine: phenotyping using dietary CMR *versus* genotyping

Clozapine and olanzapine C/D ratios decreased significantly in relation to elevated CMR. To illustrate, a patient with a CMR lower than percentile 10 (slow metabolizer) is expected to have increased C/D of 33 and 27% for clozapine and olanzapine, respectively, as compared to a normal metabolizer. A patient with a CMR higher than percentile 90 (fast metabolizer) is expected to have decreased C/D of 47 and 27% for clozapine and olanzapine, respectively. With appropriate analytical methods, CMR can be measured from the same baseline blood sample already drawn for routine hematological monitoring, allowing early assessment of CYP1A2 activity—a key determinant of personalized clozapine dose titration. This strategy parallels the recently proposed ancestry-based titration approach (ancestry serving as a proxy for CYP1A2 activity) aimed at reducing early severe adverse events such as syncope, myocarditis, and pneumonia [30]. In addition, obtaining CMR during treatment from the same sample as a clozapine or olanzapine plasma concentration measurement, would give clinicians additional information on CYP1A2 activity. This might help to distinguish a faster metabolism (CMR higher than 1.4 for clozapine) from a non-adherence problem or a slower metabolism (CMR lower than 0.9 for clozapine, or lower than 0.6 for olanzapine) from abuse. CMR explained a higher percentage of variability of clozapine C/D than the clozapine-to-norclozapine ratio, suggesting that the latter is not an ideal marker for measuring CYP1A2 activity, which is in accordance with findings from a comprehensive review [31]. In the multivariable model including only clinical factors, the influence of CMR on C/D ratios was significant for clozapine, but not for olanzapine.

In the multivariable model including genetic data, CMR was significantly associated with olanzapine C/D in Caucasians, but with a low explained variability. These results could be explained by the significant contribution of UGT1A4 in olanzapine metabolism, mitigating the impact of CYP1A2 [25]. Interaction studies with fluvoxamine, a potent CYP1A2 inhibitor, showed a 5–10-fold increase in clozapine plasma concentrations, but only a 1–2 fold increase in olanzapine concentrations, in accordance with lower involvement of CYP1A2 in olanzapine metabolism compared to clozapine [32]. In our model, rs2472297 C/T does not contribute to the variation of clozapine C/D in the Caucasian sample and explain 2.1% when all ethnicities were considered, values comparable with a reported 1.5% in a previous GWAS analysis of clozapine levels [33]. CMR explained 5.7 and 13.0% of clozapine C/D variability, in Caucasians and all ethnicities, respectively, an over six-fold higher value than for rs2472297 C/T, which is expected as CMR also captures the effect of other genetic and environmental factors.

### Association of CMR with psychiatric admission and LOS

To our knowledge, the present study is the first to link increased CYP1A2 activity (i.e., elevated CMR) with increased risks of admission and prolonged LOS in psychiatric settings. A previous study showed an increased likelihood of admission for CYP2D6 (another CYP isoform implicated in the metabolism of psychotropic drugs) ultrarapid metabolizers [34], while another study observed prolonged LOS in deficient CYP2D6 metabolizers [35]. Notably, clozapine and olanzapine were administered to only 19% of our patients during their hospital stays. However, there was no documentation of the patient’s medications prior to admission, and documentation is lacking regarding the use of other CYP1A2 substrates (e.g., duloxetine or agomelatine). Nonetheless, the associations between CMR and both admission risk and LOS observed across the full sample, suggested that these relationships were not solely driven by exposure to specific CYP1A2 substrates during the index hospitalization. This broader pattern suggests that the CMR may reflect clinically relevant variability in CYP1A2 activity that extends beyond drug metabolism alone. Given that CYP1A2 activity is influenced by other clinical (e.g., sex, smoking status, and inflammation), genetic and epigenetic factors, the CMR may serve as an integrative biomarker of individual vulnerability or treatment responsiveness. These findings underscore the potential of the CMR as a scalable and generalizable marker in psychiatric care, with relevance that extends beyond its traditional role in therapeutic drug monitoring. Future studies should further investigate the predictive value of the CMR for treatment outcomes in psychiatry and explore its utility in guiding personalized interventions.

Interestingly, none of the CMR-related SNPs was significantly associated with admission or LOS, underscoring the importance of phenotyping over genotyping for assessing CYP1A2 activity and for identifying patients at risk of treatment failure.

### Limitations

The present study has several limitations. All included studies were observational and, except for PsyMetab, all data were cross-sectional, preventing causality determination. The time between the last caffeine intake and blood sampling was only available in SKIPOGH. Although this variable accounted for 1.5% of the variance, excluding it from the multivariable analysis did not change the results, indicating that CMR is mostly independent of the time from last caffeine intake. We also could not ascertain participants’ compliance regarding drugs used as covariates (e.g., CYP1A2 inhibitors or inducers). Associations of CMR with C/D ratios of other CYP1A2 typical substrates should be tested. Moreover, the odds of admissions might be underestimated with possible admissions to other psychiatric hospitals. We cannot exclude the possibility that other unidentified or unmonitored factors may also be associated with CMR, admission, and LOS. Finally, the four cohorts included predominantly Caucasian descent; future studies must validate these associations in other populations.

## Conclusion

Random CMR, with caffeine from diet, is associated with the well-known clinical and genetic factors linked to monitored CMR that probe CYP1A2 activity. Therefore, random CMR could contribute, in addition to other clinical factors, to personalizing clozapine and, modestly, olanzapine, and possibly other CYP1A2 substrate prescriptions, and to identifying patients at risk of psychiatric admission and prolonged LOS.

## Supplementary information

Supplementary file - CYP1A2

## Data Availability

• CoLaus|PsyCoLaus The data from the CoLaus|PsyCoLaus study used in this article cannot be fully shared as they contain potentially sensitive personal information on participants. According to the Ethics Committee for Research of the Canton of Vaud, sharing these data would be a violation of Swiss legislation with respect to privacy protection. However, coded individual-level data that do not allow researchers to identify participants are available upon request to researchers who meet the criteria for data sharing of the CoLaus|PsyCoLaus Datacenter (CHUV, Lausanne, Switzerland). Any researcher affiliated with a public or private research institution who complies with the CoLaus|PsyCoLaus standards can submit a research application to research.colaus@chuv.ch or research.psycolaus@chuv.ch. Proposals requiring only baseline data will be evaluated by the baseline (local) Scientific Committee (SC) of the CoLaus and PsyCoLaus studies. Proposals requiring follow-up data will be evaluated by the follow-up (multicentric) SC of the CoLaus|PsyCoLaus cohort study. Detailed instructions for gaining access to the CoLaus|PsyCoLaus data used in this study are available at www.colaus-psycolaus.ch/professionals/how-to-collaborate/. • SKIPOGH The datasets analyzed during the current study are not publicly available due to sensitivity of the data, as it may compromise individual privacy, but may be available from Professor Murielle Bochud (main coordinator of the SKIPOGH study) upon reasonable request to murielle.bochud@unisante.ch. • PsyMetab Due to the sensitivity of the data and the absence of informed consent for public data depository, the datasets analyzed during this study are not available to the public. The dataset supporting the conclusions of this article were obtained from PsyMetab study. Requests to access the datasets should be directed to: research.psymetab@chuv.ch.
