# Supplementary material for: Dietary caffeine to assess CYP1A2 activity, tailor clozapine doses, and predict treatment response: genetic, epigenetic and clinical analyses
Source: Mol Psychiatry. 2025 Sep 17;31(3):1420–30. doi: 10.1038/s41380-025-03256-x (PMC12916320; doi:10.1038/s41380-025-03256-x)
Supplement: Supplementary file 1 — Supplementary file - CYP1A2 [file 41380_2025_3256_MOESM1_ESM.docx]

Supplementary file

Index

[Supplementary methods: 3](#_Toc198654580)

[1. Study designs, population, and setting: 3](#_Toc198654581)

[2. Genetic variability of CYP1A2 activity 6](#_Toc198654582)

[• Genome-wide association study (GWAS; CoLaus|PsyCoLaus) 6](#_Toc198654583)

[• Genetic analysis in PsyMetab cohorts 7](#_Toc198654584)

[3. Epigenome-wide association study (EWAS; SKIPOGH) 8](#_Toc198654585)

[4. Clozapine, norclozapine, olanzapine and high-sensitivity C-reactive protein plasma levels quantification 10](#_Toc198654586)

[5. Statistical analyses 12](#_Toc198654587)

[Supplementary tables 13](#_Toc198654588)

[Supplementary figures 27](#_Toc198654589)

[Supplementary Table 1: Covariate definitions 13](#_Toc158137076)

[Supplementary Table 2: Single nucleotide polymorphism (SNP) candidates potentially associated directly or indirectly with CYP1A2 activity 14](#_Toc158137077)

Supplementary Table 3: Demographic and clinical characteristics of SKIPOGH and CoLaus|PsyCoLaus cohorts………………………………………………………………………………………………….17

[Supplementary Table 4: Demographic and clinical characteristics of the clozapine pharmacokinetic and PsyMetab samples 18](#_Toc158137078)

[Supplementary Table 5: Demographic and clinical characteristics of Caucasian and non-Caucasian clozapine patients (PsyMetab) 19](#_Toc158137079)

[Supplementary Table 6: Demographic and clinical characteristics of Caucasian and non-Caucasian olanzapine patients (PsyMetab) 20](#_Toc158137080)

Supplementary Table 7: Demographic and clinical characteristics of PsyMetab patients included in hospital admission and length of stays analyses………………………………………………………..……………..21

[Supplementary Table 8: GWAS-significant SNPs associated with caffeine metabolic ratios in CoLaus|PsyCoLaus (N=3762) 22](#_Toc158137081)

Supplementary Table 9: Associations between caffeine metabolite and clozapine-to-norclozapine ratios and with clozapine…………………………………………………………………………………………………………..23

[Supplementary Table 10: Combined estimates, standard errors, and p values from 100 linear mixed-effect models fitted with imputed high-sensitivity C-reactive protein values 24](#_Toc158137082)

[Supplementary Table 11: Multivariable model including clinical factors and GWAS-significant SNPs associated with caffeine metabolic ratios as covariates in Caucasians 25](#_Toc158137083)

[Supplementary Table 12: Multivariable model including clinical factors and GWAS-significant SNPs associated with caffeine metabolic ratios as covariates in all ethnicities 26](#_Toc158137084)

[Supplementary figure 1: Factors associated with caffeine metabolic ratios in SKIPOGH considering genetics 27](#_Toc158137087)

[Supplementary figure 2: Factors associated with caffeine metabolic ratios in CoLaus|PsyCoLaus considering genetics 28](#_Toc158137088)

Supplementary figure 3: Epigenome-Wide Association Study (EWAS) results for caffeine metabolic ratios (N=565)…………………………………………………………………………………………………………………….…………….29

Supplementary figure 4: Receiver operating characteristic curve analysis evaluating the performance of CMR to classify a twofold higher and a half lower value of the median dose-normalized plasma concentrations…………………………………………………………………………………………………………………………………30

# Supplementary methods:

## Study designs, population, and setting:

- SKIPOGH

The Swiss Kidney Project on Genes in Hypertension "SKIPOGH" study is a family- and population-based study exploring genetic and environmental determinants of blood pressure.^1^ Data from the two study waves were merged together and used (SKIPOGH1 from 2009 to 2013 (N=1123) and SKIPOGH2 from 2012 to 2016 (N=931)). All participants gave written informed consent, and the Ethics Committees of Lausanne, Geneva, and Bern University Hospitals approved the study.

- CoLaus|PsyCoLaus

Data from the first physical follow-up of CoLaus|PsyCoLaus, a population-based study designed to investigate mental disorders and cardiovascular risk factors, were used.^2,3^ The first physical follow-up took place between 2009 and 2013 in a sample of 5064 participants. Only participants with caffeine and paraxanthine measurements were included (N=4898). All participants gave written informed consent, and the Ethics Committee of Lausanne approved the study.

- PsyMetab

PsyMetab is a longitudinal study that collects clinical and genetic data of psychiatric patients taking weight-inducing psychotropic medications.^4^ All participants gave written informed consent, and the Ethics Committee of Lausanne approved the study.

To investigate the association between psychiatric hospital admission and caffeine metabolic ratio, patients with or without at least one psychiatric hospital stay at Lausanne University Hospital, between January 1^st^, 2007, and June 30^th^, 2023, were selected from the PsyMetab cohort. The last available observation with exhaustive clinical data on smoking status, diagnosis, prescribed medication, and caffeine metabolic ratio within one year from the last observation date was selected (N patients with ≥1 hospital stay=664; N patients without ≥1 hospital stay= 355). For patients with at least one psychiatric hospital admission (N=664), the lengths of stay at the Department of Psychiatry of Lausanne University Hospital were calculated for each hospitalization with available clinical data on smoking status, diagnosis, prescribed medication, and caffeine metabolic ratio within one year from hospital admission or discharge (N hospitalizations=1349). Of note, hospital admissions that were interrupted for less than one day, whether voluntarily or involuntarily (e.g., patients who ran away from hospital and were readmitted the same day) were counted as a single stay.

To investigate associations between caffeine metabolic ratios, clozapine and olanzapine data were acquired from the PsyMetab study. Briefly, in- or outpatients (68 for clozapine and 146 for olanzapine) with available drug plasma levels (112 for clozapine and 222 for olanzapine), daily dosage, times of last drug intake and blood sampling, and smoking status were included.

Observations were at steady state, with blood sampling performed between 5 to 24 hours and 9 to 26 hours after last clozapine and olanzapine intake, respectively. Patients with strong CYP1A2 inhibitors (e.g, ciprofloxacin, fluvoxamine) were excluded, as well as patients with intramuscular administration of olanzapine. High-sensitivity C-reactive protein (hsCRP), caffeine and paraxanthine plasma levels were measured retrospectively.

- Clozapine pharmacokinetic study

In addition, data from a previous study on clozapine pharmacokinetics (N=75) conducted in two Swiss psychiatric clinics (Königsfelden and Lausanne) were used for clozapine analyses.^5^ All patients or their legal representatives gave written informed consent, and the Ethics Committees of the two participating centers (Königsfelden and Lausanne) approved the study.

Among the 75 adult inpatients, 52 patients were finally included after exclusion of patients with strong CYP1A2 inhibitors and CYP3A inducers (17 patients with fluvoxamine and one with phenytoin), of patients without available blood samples (4 patients) or blood sampling not performed between 5-24 hours after last clozapine intake (1 patient with a blood sampling at 36 hours). Blood samplings drawn at day 7 were used to measure hsCRP, caffeine and paraxanthine plasma levels retrospectively.

**Reference for the abovementioned study:**

1. Alwan H, Pruijm M, Ponte B, et al. Epidemiology of masked and white-coat hypertension: the family-based SKIPOGH study. *PLoS One*. 2014;9(3):e92522. doi:10.1371/journal.pone.0092522

2. Firmann M, Mayor V, Vidal PM, et al. The CoLaus study: a population-based study to investigate the epidemiology and genetic determinants of cardiovascular risk factors and metabolic syndrome. *BMC Cardiovasc Disord*. 2008;8(1):6. doi:10.1186/1471-2261-8-6

3. Preisig M, Waeber G, Vollenweider P, et al. The PsyCoLaus study: methodology and characteristics of the sample of a population-based survey on psychiatric disorders and their association with genetic and cardiovascular risk factors. BMC Psychiatry. 2009;9(1):9. doi:10.1186/1471-244X-9-9

4. Sjaarda J, Delacrétaz A, Dubath C, et al. Identification of four novel loci associated with psychotropic drug-induced weight gain in a Swiss psychiatric longitudinal study: A GWAS analysis. *Mol Psychiatry*. Published online May 12, 2023:1-8. doi:10.1038/s41380-023-02082-3

5. Jaquenoud Sirot E, Knezevic B, Morena GP, et al. ABCB1 and cytochrome P450 polymorphisms: clinical pharmacogenetics of clozapine. J Clin Psychopharmacol. 2009;29(4):319-326. doi:10.1097/jcp.0b013e3181acc372

## Genetic variability of CYP1A2 activity

## Genome-wide association study (GWAS; CoLaus|PsyCoLaus)

Genome-wide genotyping was performed using the Affymetrix Axiom array. Nuclear DNA was extracted from whole blood of all participants. Genotypes were called using BRLMM (http://www.affymetrix.com/support/technical/whitepapers/brlmm_whitepap). Duplicate individuals, and first- and second-degree relatives, were identified and then removed by computing estimates of pair-wise genomic kinship coefficients, using KING.^1^ Subjects were excluded from the analysis in case of a genotype call rate less than 95%. To account for possible population stratification, we computed principal component analysis (PCA) using PLINK^2^ with default options and excluded individuals who did not segregate with European samples using SNPweights version 2.1.^3^ Quality control for single nucleotide polymorphisms (SNPs) and insertion/deletions (indels) was performed using the following criteria: monomorphic (or with minor allele frequency (MAF) < 0.1%), call rates less than 95%, deviation from the Hardy-Weinberg equilibrium (HWE) (p < 1×10-6). Phased haplotypes were generated using eagle version 2.4.^4^ Imputation was performed using minimac4^5^ and the TOPMed Imputation Reference panel (https://www.biorxiv.org/content/10.1101/563866v1) hosted on the Michigan Imputation Server.^6^ We used imputed allele dosages for all SNPs and indels to avoid genotyping missingness. A MAF >= 0.1% and an imputation quality Rsq ≥ 0.3 was required for the inclusion of the genetic variants into further analyses.

Thus, the CMR GWAS was performed using linear regression with imputed allele dosages using EPACTS.^7^ GWAS analysis was adjusted for 5 principal components (PCAs) and the covariates listed in supplementary Table 1.

**References for GWAS:**

1. Manichaikul A, Mychaleckyj JC, Rich SS, Daly K, Sale M, Chen WM. Robust relationship inference in genome-wide association studies. *Bioinformatics*. 2010;26(22):2867-2873. doi:10.1093/bioinformatics/btq559

2. Purcell S, Neale B, Todd-Brown K, et al. PLINK: a tool set for whole-genome association and population-based linkage analyses. *Am J Hum Genet*. 2007;81(3):559-575. doi:10.1086/519795

3. Chen CY, Pollack S, Hunter DJ, Hirschhorn JN, Kraft P, Price AL. Improved ancestry inference using weights from external reference panels. *Bioinformatics*. 2013;29(11):1399-1406. doi:10.1093/bioinformatics/btt144

4. Loh PR, Danecek P, Palamara PF, et al. Reference-based phasing using the Haplotype Reference Consortium panel. *Nat Genet*. 2016;48(11):1443-1448. doi:10.1038/ng.3679

5. Fuchsberger C, Abecasis GR, Hinds DA. minimac2: faster genotype imputation. *Bioinformatics*. 2015;31(5):782-784. doi:10.1093/bioinformatics/btu704

6. Das S, Forer L, Schönherr S, et al. Next-generation genotype imputation service and methods. *Nat Genet*. 2016;48(10):1284-1287. doi:10.1038/ng.3656

7. EPACTS - Genome Analysis Wiki. Accessed August 7, 2023. <https://genome.sph.umich.edu/wiki/EPACTS>

## Genetic analysis in PsyMetab cohorts

All patients were genotyped on a Infinium Global Screening Array v2 at the Genomics Platform of iGE3 in Geneva, Switzerland, as described elsewhere.^1^ SNPs significantly associated with CMR in the CoLaus|PsyCoLaus GWAS were tested in all the PsyMetab cohorts.

**References for PsyMetab genetic analyses**

1. Sjaarda J, Delacrétaz A, Dubath C, et al. Identification of four novel loci associated with psychotropic drug-induced weight gain in a Swiss psychiatric longitudinal study: A GWAS analysis. *Mol Psychiatry*. Published online May 12, 2023:1-8. doi:10.1038/s41380-023-02082-3

## Epigenome-wide association study (EWAS; SKIPOGH)

Epigenome-wide DNA methylation from white blood cells was measured in 250 SKIPOGH 2 participants (the second study wave) using the Infinium Human Methylation 450 BeadChip microarray of Illumina (HM450: 485,512 CpG sites). For a different set of 451 SKIPOGH 2 participants, epigenome-wide DNA methylation was measured using a more recent Infinium MethylationEPIC v1.0 microarray (EPIC), including >90% of the CpG sites from the HM450 and an additional 413,743 CpGs (865,859 CpGs in total).^1^ In the present analyses, 452’453 CpG sites available across both arrays were used. Specific details on CpG data pre-processing (accounting for technical nuisance, imputation of missing values, M-values calculation) have been described elsewhere.^2,3^

For statistical analyses, we implemented fixed-effect linear regression models for the EWAS analysis between 452’453 CpG markers as a combined set of dependent variables, and CMR, as successive independent variables, using data from 565 SKIPOGH2 participants (136 excluded due to missing values for covariates). The regression models were adjusted for age, sex, recruitment center (Lausanne, Geneva, Bern), seasonality of blood sampling (spring, summer, fall, winter), chip type (HM450, EPIC), CPACOR principal components for technical nuisance (n=30), Houseman-estimated white blood cell composition (CD8, CD4, NK, B cells, Monocytes, Granulocytes), BMI, alcohol consumption, statin, hypertension status, time interval between blood drawing and last caffeine intakes, CYP1A2 inducers, as well as rs4410790, rs59251770, rs2472297, and rs56113850 genotypes. The obtained EWAS results were presented using Manhattan plot, while Bonferroni and Benjamini-Hochberg (BH-False Discovery Rate) methods were applied for multiple testing.

**Reference for the EWAS**

1. Pidsley R, Zotenko E, Peters TJ, et al. Critical evaluation of the Illumina MethylationEPIC BeadChip microarray for whole-genome DNA methylation profiling. Genome Biol. 2016;17(1):208. doi:10.1186/s13059-016-1066-1

2. Ghobril JP, Petrovic D, Ehret G, et al. PhenoExplorer: An Interactive Web-based Platform for Exploring (Epi)Genome-Wide Associations Using a Swiss Population-based Study. CHIMIA. 2022;76(12):1052-1052. doi:10.2533/chimia.2022.1052

3. Petrovic D, Carmeli C, Sandoval JL, et al. Life-course socioeconomic factors are associated with markers of epigenetic aging in a population-based study. *Psychoneuroendocrinology*. 2023;147:105976. doi:10.1016/j.psyneuen.2022.10597

## Clozapine, norclozapine, olanzapine and high-sensitivity C-reactive protein plasma levels quantification

Clozapine, norclozapine and olanzapine plasma levels were quantified during routine TDM. Blood samples were collected in EDTA-containing tubes, centrifugated and stored at -20°C until analysis (i.e., 1-4 days after blood sampling) in an accredited environment (ISO 15189). For PsyMetab study, clozapine and norclozapine were quantified by high performance liquid chromatography (Agilent Series 1100 LC System) coupled to mass spectrometry (Agilent Series 1100 MSD) with limits of quantification of 2 ng/mL^1^ or UHPLC (Waters ACQUITY UPLC system)-MS/MS (Waters Xevo TQ-XS) with limits of quantification of 1 ng/mL (detailed method available on request). For the clozapine pharmacokinetic study, a gas chromatography with a nitrogen-phosphorus detector method was used, with limits of quantification of 4 ng/ml for both analytes.^2^ Olanzapine plasma quantification was performed by UHPLC (Waters ACQUITY UPLC system)-MS/MS (Waters TQD or Waters Xevo TQ-S) with a limit of quantification of 0.5 ng/mL.^3^ Finally, high- sensitive C-reactive proteins were determined by an immunoassay on a Cobas integra 400 plus Roche® analyzer (Roche Diagnostics, Rotkreuz, Switzerland) for clozapine samples and an Indiko plus Thermo Scientific® analyzer (Thermo Fisher Scientific, Reinach, Switzerland) for olanzapine samples.

Assuming that the missing values in high-sensitivity C-reactive protein (hsCRP) were at random, data was imputed using chain equation generating 100 multiple copies of datasets. The imputation process included the outcome and co-variates used in the multivariable model (no missing data except for hsCRP). Linear mixed models were applied on each imputed dataset, estimates, standard error, and P-values were summarized using Rubin’s rules. Sensitivity analyses were used to assert the missing at randomness in hsCRP, by conducting the same analyses in non-imputed dataset and comparing the results.

**Reference for Clozapine, norclozapine and olanzapine plasma levels assessments**

1. Choong E, Rudaz S, Kottelat A, Guillarme D, Veuthey JL, Eap CB. Therapeutic drug monitoring of seven psychotropic drugs and four metabolites in human plasma by HPLC-MS. J Pharm Biomed Anal. 2009 Dec 5;50(5):1000-8. doi: 10.1016/j.jpba.2009.07.007.

2. Jaquenoud Sirot E, Knezevic B, Morena GP, et al. ABCB1 and cytochrome P450 polymorphisms: clinical pharmacogenetics of clozapine. J Clin Psychopharmacol. 2009;29(4):319-326. doi:10.1097/jcp.0b013e3181acc372.

3. Ansermot N, Brawand-Amey M, Kottelat A, Eap CB. Fast quantification of ten psychotropic drugs and metabolites in human plasma by ultra-high performance liquid chromatography tandem mass spectrometry for therapeutic drug monitoring. J Chromatogr A. 2013 May 31;1292:160-72. doi: 10.1016/j.chroma.2012.12.071.

## Statistical analyses

- **CoLaus|PsyCoLaus full clinical model formula**

bc<-boxcox(lm(CMR ~ Inducers + logcrp + hypertension +alcohol +I(age/10) +sex + I(eGFR/10) + statin+ bmi , data=data))

lambda <- bc$x[which.max(bc$y)]

with(data,hist(CMR^lambda))

Full.model<-lm((CMR^(lambda)-1)/lambda ~ Inducers + logcrp + hypertension +alcohol +I(age/10) +sex + bmi, data=data, na.action=na.omit)

- **SKIPOGH full clinical model formula**

lambda <- 0.18888888

Full.model<-lmer((CMR^(lambda)-1)/lambda ~ Inducers + logcrp + hypertension + alcohol + I(age/10) + I(eGFR/10) + sex + statin + time +(1|Family_ID/Participant_ID), data=data,na.action=na.omit)

# Supplementary tables

Supplementary Table 1: Covariate definitions

| **Covariates** | **Definition** | |
| --- | --- | --- |
|  | **SKIPOGH** | **CoLaus\|PsyCoLaus** |
| Age | Years | |
| Sex | Male, female | |
| Body mass index | Weight (kilograms)/ Height^2^ (meters) | |
| Smoking status | Currently smoking (self-reported) | |
| Alcohol consumption | Present consumption of alcohol (self-reported) | At least one drink per week (self-reported) |
| eGFR‡ | Chronic Kidney Disease - Epidemiology Collaboration (CKD-EPI) formula (milliliter/minute/1.73 meter^2^) | |
| CYP1A2 activity inhibitors§ | [Amiodarone](https://compendium.ch/fr/product/1003487-amiodar-cpr-200-mg) [/ [Atazanavir](https://compendium.ch/fr/product/1367701-atazanavir-mepha-caps-200-mg) / Cannabinoids](https://compendium.ch/fr/product/1254995-sativex-sol) / [Ciprofloxacin](https://compendium.ch/fr/product/108880-cip-eco-cpr-pell-250-mg) / Ethinylestradiol / [Fluoxetine](https://compendium.ch/fr/product/26667-fluctine-caps-20-mg) / [Fluvoxamine](https://compendium.ch/fr/product/25002-floxyfral-cpr-pell-100-mg) / [Moclobemide](https://compendium.ch/fr/product/2635-aurorix-cpr-pell-150-mg) / [Norfloxacine](https://compendium.ch/fr/product/76539-norsol-cpr-400-mg) / [Paroxetine](https://compendium.ch/fr/product/34860-deroxat-cpr-pell-20-mg) / [Propafenone](https://compendium.ch/fr/product/9986-rytmonorm-cpr-pell-150-mg) / [Sertraline](https://compendium.ch/fr/product/1026596-sertragen-cpr-pell-50-mg) / [Quetiapine](https://compendium.ch/fr/product/1199394-quetiapin-mepha-cpr-pell-25-mg) / Tipranavir / [Verapamil](https://compendium.ch/fr/product/3905-isoptin-cpr-pell-80-mg) | |
| CYP1A2 activity inducers§ | Smoking or one or more of the following drugs: [Carbamazepine](https://compendium.ch/fr/product/33128-tegretol-cpr-200-mg) / [Esomeprazole](https://compendium.ch/fr/product/1152179-esomep-i-v-subst-seche-40-mg) / [Lansoprazole](https://compendium.ch/fr/product/54877-agopton-caps-15-mg) / [Modafinil](https://compendium.ch/fr/product/1162431-modasomil-cpr-100-mg) / [Omeprazole](https://compendium.ch/fr/product/1323823-omed-antacid-sandoz-caps-20-mg) / [Ritonavir](https://compendium.ch/fr/product/1157702-norvir-cpr-pell-100-mg) | |
| Statins⁞ | HMG CoA reductase inhibitors (ATC codes: C10AA) | |
| Hypertension | Blood pressure>140/90 millimeter of mercury, self-report, or taking anti-hypertensive drugs | |
| High-sensitivity C-reactive protein | Immunoassay and latex HS (Roche Diagnostics, Switzerland) | Immunoassay and latex HS (IMMULITE 1000-High) |
| Time | Time between blood sampling and last caffeine intake in hours | Not available |

‡Measured using the method referenced in: Levey AS, Stevens LA, Schmid CH, et al. A New Equation to Estimate Glomerular Filtration Rate. *Ann Intern Med*. 2009;150(9):604-612

§ Selected using: CYP P450 Drug Interactions Service de pharmacologie et toxicologie clinique; Hôpitaux Universitaires Genève. Accessed January 14, 2021. https://www.hug.ch/sites/interhug/files/structures/pharmacologie_et_toxicologie_cliniques/carte_cytochromes_2016_final.pdf

**⁞**Selected using: WHOCC - ATC/DDD Index: <https://www.whocc.no/atc_ddd_index/?code=C10AA&showdescription=no>. Accessed January 14, 2021

Abbreviation: eGFR= estimated Glomerular Filtration Rate

Supplementary Table 2: Single nucleotide polymorphism (SNP) candidates potentially associated directly or indirectly with CYP1A2 activity

| **Nearest genes** | **Single nucleotide polymorphisms** | **Nearest genes** | **Single nucleotide polymorphisms** |
| --- | --- | --- | --- |
| *AHR* | rs4410790^1–4^ | *TCF1 (HNF1α)* | rs2464196^5^ |
|  | rs2282885^5^ |  | rs1169306^5^ |
|  | rs7811989^5^ | *NCOA1 (SRC1)* | rs2119115^5^ |
|  | rs2066853^5^ | *IL1β* | rs1143634^5^ |
|  | rs10275488^3^ | NR1I1 (VDR) | rs1540339^5^ |
|  | rs2892838^3^ |  | rs2228570^5^ |
|  | rs117692895^6^ | *NR1I2 (PXR)* | rs1523130^5^ |
|  | rs4719497^6^ | *NR1I3 (CAR)* | rs2502815^5^ |
|  | rs11766104^7^ |  | rs4073054^5^ |
|  | rs7791070^7^ | *NR2B1 (RXRα)* | rs3818740^5^ |
|  | rs2134688^5^ |  | rs3132297^5^ |
| *AHRR* | rs2241598^5^ | *CYP1A2* | rs1133323^8^ |
| *POR* | rs2302429^9^ |  |  |
|  | rs10239977^9^ |  | rs12909047^3^ |
|  | rs2286823^9^ |  | rs62005807^3^ |
|  | rs1057868^9^ |  |  |
| *CYP1A1/CYP1A2* | rs2470893^1–3,10^ |  | rs762551 (CYP1A2*1F)^1,11–17^ |
|  | rs2472297^1,3,4,10,18,19^ |  |  |

The references are indicated in superscript numbers.

**REFERENCES OF SNPs CANDIDATES**

1. Söderberg MM, Haslemo T, Molden E, Dahl ML. Influence of CYP1A1/CYP1A2 and AHR polymorphisms on systemic olanzapine exposure. *Pharmacogenet Genomics*. 2013;23(5):279-285. doi:10.1097/FPC.0b013e3283602876

2. Cornelis MC, Monda KL, Yu K, et al. Genome-Wide Meta-Analysis Identifies Regions on 7p21 (AHR) and 15q24 (CYP1A2) As Determinants of Habitual Caffeine Consumption. *PLOS Gen*. 2011;7(4):e1002033. doi:10.1371/journal.pgen.1002033

3. Cornelis MC, Kacprowski T, Menni C, et al. Genome-wide association study of caffeine metabolites provides new insights to caffeine metabolism and dietary caffeine-consumption behavior. *Hum Mol Genet*. 2016;25(24):5472-5482. doi:10.1093/hmg/ddw334

4. Söderberg MM, Molden E, Dahl ML. No influence of CYP3A43 rs472660G> A on steady-state serum olanzapine concentrations in white psychiatric patients. *Pharmacogenet Genomics*. 2014;24(5):272-275. doi:10.1097/FPC.0000000000000041

5. Dobrinas M, Cornuz J, Eap CB. Pharmacogenetics of CYP1A2 activity and inducibility in smokers and exsmokers. *Pharmacogenet Genomics*. 2013;23(5):286-292. doi:10.1097/FPC.0b013e3283602e75

6. Zhong VW, Kuang A, Danning RD, et al. A genome-wide association study of bitter and sweet beverage consumption. *Hum Mol Genet*. 2019;28(14):2449-2457. doi:10.1093/hmg/ddz061

7. Kennedy OJ, Pirastu N, Poole R, et al. Coffee Consumption and Kidney Function: A Mendelian Randomization Study. *Am J Kidney Dis*. 2020;75(5):753-761. doi:10.1053/j.ajkd.2019.08.025

8. Guessous I, Dobrinas M, Kutalik Z, et al. Caffeine intake and CYP1A2 variants associated with high caffeine intake protect non-smokers from hypertension. *Hum Mol Genet*. 2012;21(14):3283. doi:10.1093/hmg/dds137

9. Dobrinas M, Cornuz J, Pedrido L, Eap CB. Influence of cytochrome P450 oxidoreductase genetic polymorphisms on CYP1A2 activity and inducibility by smoking. *Pharmacogenet Genomics*. 2012;22(2):143-151. doi:10.1097/FPC.0b013e32834e9e1a

10. Amin N, Byrne E, Johnson J, et al. Genome-wide association analysis of coffee drinking suggests association with CYP1A1/CYP1A2 and NRCAM. *Mol Psychiatry*. 2012;17(11):1116-1129. doi:10.1038/mp.2011.101

11. Czerwensky F, Leucht S, Steimer W. CYP1A2*1D and *1F polymorphisms have a significant impact on olanzapine serum concentrations. *Ther Drug Monit*. 2015;37(2):152-160. doi:10.1097/FTD.0000000000000119

12. Laika B, Leucht S, Heres S, Schneider H, Steimer W. Pharmacogenetics and olanzapine treatment: CYP1A2*1F and serotonergic polymorphisms influence therapeutic outcome. *Pharmacogenomics J*. 2010;10(1):20-29. doi:10.1038/tpj.2009.32

13. Koonrungsesomboon N, Khatsri R, Wongchompoo P, Teekachunhatean S. The impact of genetic polymorphisms on CYP1A2 activity in humans: a systematic review and meta-analysis. *Pharmacogenomics J*. 2018;18(6):760-768. doi:10.1038/s41397-017-0011-3

14. Dobrinas M, Cornuz J, Oneda B, Kohler Serra M, Puhl M, Eap C. Impact of Smoking, Smoking Cessation, and Genetic Polymorphisms on CYP1A2 Activity and Inducibility. *CPT*. 2011;90(1):117-125. doi:10.1038/clpt.2011.70

15. Gunes A, Ozbey G, Vural EH, et al. Influence of genetic polymorphisms, smoking, gender and age on CYP1A2 activity in a Turkish population. *Pharmacogenomics*. 2009;10(5):769-778. doi:10.2217/pgs.09.22

16. Na Takuathung M, Hanprasertpong N, Teekachunhatean S, Koonrungsesomboon N. Impact of CYP1A2 genetic polymorphisms on pharmacokinetics of antipsychotic drugs: a systematic review and meta-analysis. *Acta Psychiatr Scand*. 2019;139(1):15-25. doi:10.1111/acps.12947

17. Saiz-Rodríguez M, Ochoa D, Belmonte C, et al. Polymorphisms in CYP1A2, CYP2C9 and ABCB1 affect agomelatine pharmacokinetics. *J Psychopharmacol*. 2019;33(4):522-531. doi:10.1177/0269881119827959

18. Sulem P, Gudbjartsson DF, Geller F, et al. Sequence variants at CYP1A1-CYP1A2 and AHR associate with coffee consumption. *Hum Mol Genet*. 2011;20(10):2071-2077. doi:10.1093/hmg/ddr086

19. Pardiñas AF, Nalmpanti M, Pocklington AJ, et al. Pharmacogenomic Variants and Drug Interactions Identified Through the Genetic Analysis of Clozapine Metabolism. *Am J Psychiatry*. 2019;176(6):477-486. doi:10.1176/appi.ajp.2019.18050589

**Supplementary Table 3:** Demographic and clinical characteristics of SKIPOGH and CoLaus|PsyCoLaus cohorts

|  | **SKIPOGH (N=2054)** | **CoLaus\|PsyCoLaus (N=4898)** | **p-value** |
| --- | --- | --- | --- |
| **Age** (years; median [IQR])  Range (min-max) | 50 [34; 63]  (17-96) | 57 [49; 66]  (40-82) | **<10^-4^** |
| **Sex** (N (%))  Male | 974 (47) | 2609 (53) | 0.59 |
| **Ethnicity** (Caucasian; N (%))  Yes  No | 2030 (99)  24 (1) | 4519 (92)  379 (8) | **<10^-3^** |
| **Current smokers** (N (%))  Yes  No  Unknown | 507 (25)  1531 (75)  16 | 1060 (22)  3792 (78)  46 | **0.006** |
| **Alcohol consumers** (N (%))  Yes  No  Unknown | 1346 (66)  688 (34)  20 | 3653 (75)  1245 (25) | **<10^-3^** |
| **BMI** (kg/m^2^; median [IQR])  Range (min-max) | 24.5 [21.9; 27.7]  (13.8-50.1) | 25.6 [23.0; 28.6]  (14.2-54.2) | **<10^-4^** |
| **eGFR** (ml/min/1,73 m²; median [IQR])  Range (min-max) | 96 [83; 107]  (19-151) | 83 [73; 95]  (7-186) | **<10^-4^** |
| **Hypertension** (N (%))  Yes  No | 597 (29)  1439 (71) | 2030 (42)  2861 (58) | **<10^-3^** |
| **Statins** (N (%))  Yes  No  Unknown | 199 (10)  1842 (90)  13 | 805 (16)  4093 (84) | **<10^-3^** |
| **High sensitivity C-reactive protein** (mg/L; median [IQR])  Range (min-max) | 1.0 [0.5; 2.3]  (0.3-69) | 1.3 [0.6; 2.5]  (0.1-10) | 0.25 |
| **CYP1A2 activity inhibitors** (N (%))  Yes  No | 48 (2)  2006 (98) | 191 (4)  4707 (96) | **0.001** |
| **CYP1A2 activity inducers**‡ (N (%))  Yes  No | 556 (27)  1498 (73) | 1284 (26)  3614 (74) | 0.46 |
| **Caffeine metabolic ratios** (median [IQR])§  Range (min-max) | 1.2 [0.8; 1.8]  (0.1-7) | 1.2 [0.8; 1.7]  (0.03-20) | 0.91 |

Comparison between both cohorts was conducted using Pearson χ2 or Wilcoxon tests, depending on the variable types and considering the hierarchical data structure for SKIPOGH study.

Statistically significant p-values in bold.

‡ Including smokers. § Calculated by dividing paraxanthine by caffeine plasma levels and multiplying the ratio by 1.07769 (Molecular mass ratio).

Abbreviations: BMI=body mass index; eGFR=estimated Glomerular Filtration Rate; IQR=interquartile range; kg=kilograms; L=liter; m^2^=square meter; mg=milligram; min=minimum; max=maximum; mL=milliliter; N=number.

Supplementary Table 4: Demographic and clinical characteristics of the clozapine pharmacokinetic and PsyMetab samples

| **Baseline characteristics** | **Overall** | **Clozapine pharmacokinetic study** | **PsyMetab** | **p-value** |
| --- | --- | --- | --- | --- |
|  | **N = 120** | **N = 52** | **N = 68** |  |
| **Age** (years; median [IQR]) | 49 [37, 67] | 46 [39, 61] | 51 [33, 73] | 0.82 |
| Range (min-max) | (17-90) | (21-90) | (17-87) |  |
| **Sex** (N (%)) |  |  |  | 0.96 |
| Male | 62 (52%) | 27 (52%) | 35 (51%) |  |
| **Current smokers** (N (%)) | 55 (46%) | 29 (56%) | 26 (38%) | 0.056 |
| **BMI** (kg/m2; median [IQR]) | 25.4 [23.1, 29.1] | 27.5 [24.2, 30.1] | 24.4 [22.5, 28.4] | 0.012 |
| Range (min-max) | (14.2-37.5) | (19.1-35.9) | (14.2-37.5) |  |
| **Observational characteristics** | **N = 164** | **N = 52** | **N = 112** |  |
| **Daily dose** (mg/day; median [IQR]) | 300 [150, 400] | 350 [200, 506] | 263 [119, 363] | **0.006** |
| Range (min-max) | (13-900) | (25-800) | (13-900) |  |
| **Drug dosage regimen** |  |  |  | **0.003** |
| 1 time daily | 59 (36%) | 12 (23%) | 47 (42%) |  |
| 2 times daily | 74 (45%) | 22 (42%) | 52 (46%) |  |
| 3 times daily | 19 (12%) | 12 (23%) | 7 (6.3%) |  |
| 4 times daily | 12 (7.3%) | 6 (12%) | 6 (5.4%) |  |
| **Time between drug intake and blood sampling** (hours; median [IQR]) | 13.1 [11.0, 14.0] | 10.8 [10.5, 13.5] | 13.5 [12.0, 14.3] | **<0.001** |
| Range (min-max) | (5.5-24.0) | (9.2-21.0) | (5.5-24.0) |  |
| **Clozapine plasma concentration** (ng/mL; median [IQR]) | 282 [139, 460] | 356 [156, 521] | 252 [117, 414] | 0.10 |
| Range (min-max) | (8-1140) | (15-1140) | (8-879) |  |
| **Clozapine plasma concentration / dose ratios** ((ng/ml)/mg; median [IQR]) | 1.12 [0.69, 1.53] | 1.02 [0.55, 1.34] | 1.15 [0.75, 1.58] | 0.14 |
| Range (min-max) | (0.08-5.36) | (0.33-2.86) | (0.08-5.36) |  |
| **Norclozapine plasma concentration** (ng/mL; median [IQR]) | 148 [67, 259] | 144 [78, 262] | 150 [63, 259] | 0.78 |
| Range (min-max) | (7-607) | (15-603) | (7-607) |  |
| **Clozapine / norclozapine plasma concentration ratios** (median [IQR]) | 1.71 [1.41, 2.23] | 1.97 [1.47, 2.50] | 1.66 [1.41, 2.02] | **0.045** |
| Range (min-max) | (0.72-8.47) | (0.89-8.47) | (0.72-3.94) |  |
| **Caffeine metabolic ratios** ‡ (median [IQR]) | 1.1 [0.8, 1.9] | 1.2 [0.8, 2.2] | 1.0 [0.7, 1.7] | 0.09 |
| Range (min-max) | (0.2-4.5) | (0.4-4.1) | (0.2-4.5) |  |
| **High sensitivity C-reactive protein** (mg/L; median [IQR]) Range (min-max) | 2.3 [0.9, 5.1] | 2.0 [0.9, 4.5] | 2.6 [0.9, 5.1] | 0.68 |
| Range (min-max) | (0.2-136.4) | (0.2-53.0) | (0.2-136.4) |  |
| Unknown | 52 | 0 | 52 |  |

comparison between the two groups was conducted using Pearson χ2 or Wilcoxon tests, depending on the variable types.

Statistically significant p-values in bold.

‡ Calculated by dividing paraxanthine by caffeine plasma levels and multiplying the ratio by 1.07769 (Molecular mass ratio).

Abbreviations: BMI=body mass index; IQR=interquartile range; kg=kilograms; L=liter; m^2^=square meter; mg=milligram; min=minimum; max=maximum; mL=milliliter; N=number; ng=nanogram.

Supplementary Table 5: Demographic and clinical characteristics of Caucasian and non-Caucasian clozapine patients (PsyMetab)

| **Baseline characteristics** | **Overall** | **Caucasian** | **non-Caucasian** | **p-value** |
| --- | --- | --- | --- | --- |
|  | **N = 62** | **N = 56** | **N = 6** |  |
| **Age** (Years; median [IQR]) | 53 [34, 74] | 58 [41, 74] | 22 [19, 31] | **0.001** |
| Range (min-max) | (17-87) | (18-87) | (17-37) |  |
| **Sex** (N (%)) |  |  |  | 0.67 |
| Male | 32 (52%) | 28 (50%) | 4 (67%) |  |
| **Current smokers** (N (%)) | 22 (35%) | 19 (34%) | 3 (50%) | 0.66 |
| **BMI** (kg/m2; median [IQR]) | 24.4 [22.5, 27.7] | 24.4 [22.4, 27.8] | 24.6 [23.4, 27.1] | 0.82 |
| Range (min-max) | (14.2-37.5) | (14.2-37.5) | (19.9-32.9) |  |
| **Observational characteristics** | **N = 103** | **N = 95** | **N = 8** |  |
| **Daily dose** (mg/day; median [IQR]) | 275 [113, 375] | 250 [100, 350] | 300 [300, 400] | 0.16 |
| Range (min-max) | (13-900) | (13-900) | (150-450) |  |
| **Drug dosage regimen** |  |  |  | 0.9 |
| 1 time daily | 42 (41%) | 39 (41%) | 3 (38%) |  |
| 2 times daily | 49 (48%) | 44 (46%) | 5 (63%) |  |
| 3 times daily | 6 (5.8%) | 6 (6.3%) | 0 (0%) |  |
| 4 times daily | 6 (5.8%) | 6 (6.3%) | 0 (0%) |  |
| **Time between drug intake and blood sampling** (hours; median [IQR]) | 13.50 [12.00, 14.00] | 13.50 [12.38, 14.25] | 11.63 [9.88, 12.13] | **0.003** |
| Range (min-max) | (5.50-24.00) | (5.50-24.00) | (9.50-14.00) |  |
| **Plasma concentration** (ng/mL; median [IQR]) | 243 [116, 412] | 243 [124, 406] | 183 [99, 485] | 0.74 |
| Range (min-max) | (21-879) | (21-879) | (25-599) |  |
| **Plasma concentration / dose ratios** ((ng/ml)/mg; median [IQR]) | 1.15 [0.77, 1.52] | 1.18 [0.80, 1.58] | 0.76 [0.33, 1.17] | 0.06 |
| Range (min-max) | (0.08-5.36) | (0.11-5.36) | (0.08-2.00) |  |
| **Norclozapine plasma concentration** (ng/mL; median [IQR]) | 148 [63, 259] | 151 [63, 257] | 88 [68, 273] | 0.67 |
| Range (min-max) | (10-607) | (16-607) | (10-320) |  |
| **Clozapine norclozapine plasma concentration ratios** (median [IQR]) | 1.65 [1.41, 2.05] | 1.64 [1.38, 1.98] | 1.87 [1.66, 2.19] | 0.23 |
| Range (min-max) | (0.72-3.94) | (0.72-3.94) | (1.41-2.50) |  |
| **Caffeine metabolic ratios** ‡ (median [IQR]) | 1.0 [0.8, 1.7] | 1.0 [0.8, 1.6] | 2.5 [1.0, 3.7] | **0.04** |
| Range (min-max) | (0.2-4.4) | (0.2-4.4) | (0.3-4.0) |  |
| **High sensitivity C-reactive protein** (mg/L; median [IQR]) Range (min-max) | 2.8 [0.9, 5.1] | 2.8 [0.9, 5.1] | 2.1 [1.0, 4.7] | 0.99 |
| Range (min-max) | (0.3-136.4) | (0.3-136.4) | (0.9-8.8) |  |
| Unknown | 48 | 44 | 4 |  |

Comparison between the two groups was conducted using Pearson χ2 or Wilcoxon tests, depending on the variable types.

Statistically significant p-values in bold.

‡ Calculated by dividing paraxanthine by caffeine plasma levels and multiplying the ratio by 1.07769 (Molecular mass ratio).

Abbreviations: BMI=body mass index; IQR=interquartile range; kg=kilograms; L=liter; m^2^=square meter; mg=milligram; min=minimum; max=maximum; mL=milliliter; N=number; ng=nanogram.

Supplementary Table 6: Demographic and clinical characteristics of Caucasian and non-Caucasian olanzapine patients (PsyMetab)

| **Baseline characteristics** | **Overall** | **Caucasian** | **non-Caucasian** | **p-value** |
| --- | --- | --- | --- | --- |
|  | **N = 139** | **N = 98** | **N = 41** |  |
| **Age** (Years; median [IQR]) | 40 [28, 55] | 43 [31, 61] | 31 [24, 47] | **<0.001** |
| Range (min-max) | (13-90) | (13-90) | (14-66) |  |
| **Sex** (N (%)) |  |  |  | 0.83 |
| Male | 76 (55%) | 53 (54%) | 23 (56%) |  |
| **Current smokers** (N (%)) | 71 (51%) | 45 (46%) | 26 (63%) | 0.06 |
| **BMI** (kg/m2; median [IQR]) | 24.2 [21.7, 27.6] | 24.1 [21.9, 27.5] | 24.6 [21.2, 27.7] | 0.89 |
| Range (min-max) | (13.7-42.9) | (15.1-42.9) | (13.7-34.8) |  |
| **Observational characteristics** | **N = 210** | **N = 143** | **N = 67** |  |
| **Daily dose** (mg/day; median [IQR]) | 15 [10, 20] | 15 [9, 20] | 20 [10, 20] | 0.13 |
| Range (min-max) | (3-35) | (3-35) | (3-30) |  |
| **Time between drug intake and blood sampling** (hours; median [IQR]) | 13.5 [12.0, 14.5] | 13.5 [12.0, 14.7] | 13.5 [11.8, 14.1] | 0.4 |
| Range (min-max) | (9.0-26.0) | (9.0-26.0) | (9.0-24.0) |  |
| **Plasma concentration** (ng/mL; median [IQR]) | 27 [14, 42] | 28 [15, 45] | 24 [15, 38] | 0.33 |
| Range (min-max) | (1-138) | (4-138) | (1-128) |  |
| **Plasma concentration / dose ratios** ((ng/ml)/mg; median [IQR]) | 2.06 [1.50, 2.93] | 2.40 [1.60, 3.20] | 1.70 [1.27, 2.24] | **<0.001** |
| Range (min-max) | (0.05-6.40) | (0.25-6.40) | (0.05-6.40) |  |
| **Caffeine metabolic ratios** ‡ (median [IQR]) | 1.1 [0.7, 2.0] | 1.0 [0.7, 1.9] | 1.5 [0.9, 2.4] | **0.03** |
| Range (min-max) | (0.1-6.2) | (0.1-5.7) | (0.2-6.2) |  |
| **High sensitivity C-reactive protein** (mg/L; median [IQR]) Range (min-max) | 2.3 [1.0, 5.3] | 1.9 [0.9, 3.7] | 2.8 [1.3, 5.7] | 0.23 |
| Range (min-max) | (0.1-50.3) | (0.2-50.3) | (0.1-49.4) |  |
| Unknown | 80 | 56 | 24 |  |

Comparison between the two groups was conducted using Pearson χ2 or Wilcoxon tests, depending on the variable types.

Statistically significant p-values in bold.

‡ Calculated by dividing paraxanthine by caffeine plasma levels and multiplying the ratio by 1.07769 (Molecular mass ratio).

Abbreviations: BMI=body mass index; IQR=interquartile range; kg=kilograms; L=liter; m^2^=square meter; mg=milligram; min=minimum; max=maximum; mL=milliliter; N=number; ng=nanogram.

Supplementary Table 7: Demographic and clinical characteristics of PsyMetab patients included in hospital admission and length of stay analyses

| **Baseline characteristics** | **Overall**  **N=1019** |
| --- | --- |
|  |  |
| **Age** (Years; median [IQR]) | 43 [31, 57] |
| Range (min-max) | (14 - 92) |
| **Sex** (N (%)) |  |
| Male | 540 (53%) |
| **Current smokers** (N (%)) | 524 (51%) |
| **Psychiatric diagnosis** §(N (%)) |  |
| Others  Mood disorders  Psychotic disorders | 194 (19%)  414 (41%)  411 (40%) |
| **Hospital admission¦** (N (%))  Yes | 664 (65%) |
| **Clozapine or olanzapine use** (N (%)) | 159 (16%) |
| **Caffeine metabolic ratios** ‡ (median [IQR])  Range (min-max) | 1.2 [0.7,1.9]  (0.04 – 7.0) |
| **Observational characteristics** | **(N=1349)** |
| **Length of stay** (days; median [IQR]) | 42 [22, 77] |
| Range (min-max) | (1-1311) |
| **Clozapine or olanzapine use during the hospital stay** (N (%)) | 262 (19%) |
| **Caffeine metabolic ratios** ‡ (median [IQR]) | 1.2 [0.7,1.9] |
| Range (min-max) | (0.04-6.5) |

§ Psychiatric diagnosis were defined as follows: psychotic disorders [F20-F25 and F28-F29], mood disorders [F30-F31 and F32-F33], and other diagnoses [F00-F19 and F34- F99].

¦ At least one hospital admission.

‡ Calculated by dividing paraxanthine by caffeine plasma levels and multiplying the ratio by 1.07769 (Molecular mass ratio).

Abbreviations: IQR=interquartile range; min=minimum; max=maximum; N=number.

Supplementary Table 8: GWAS-significant SNPs associated with caffeine metabolic ratios in CoLaus|PsyCoLaus (N=3762)

| CHR | Position | SNP | A1 | A2 | MAF | p-value | Beta | SE | Nearest gene |
| --- | --- | --- | --- | --- | --- | --- | --- | --- | --- |
| 7 | 17244953 | rs4410790 | C | T | 0.39673 | 1.29E-19 | 0.20296 | 0.022276 | AHR |
| 7 | 152992887 | rs59251770 | A | G | 0.083665 | 7.12E-08 | 0.21004 | 0.038903 | ACTR3B |
| 15 | 74735539 | rs2472297 | T | C | 0.18438 | 3.59E-20 | 0.25376 | 0.027426 | CYP1A1-CYP1A2 |
| 19 | 40847202 | rs56113850 | C | T | 0.43856 | 5.10E-17 | -0.18925 | 0.022464 | CYP2A6 |

Abbreviations: The chromosome (Chr) and basepair position is given with regards to the GRCh38 assembly. SNP: single nucleotide polymorphism. A1/A2 represent the effect allele and the non-effect allele. MAF: minor allele frequency. *Effect size (Beta) and standard error (SE) reported for the A1 allele.*

**Supplementary Table 9:** Associations between caffeine metabolite and clozapine-to-norclozapine ratios with clozapine plasma concentration-to-dose ratios.

|  | **Clozapine [ng/mL] / dose** | | | **Clozapine [ng/mL] / dose** | | |
| --- | --- | --- | --- | --- | --- | --- |
|  | **[mg/d]^1^** | | | **[mg/d]^1^** | | |
| *Predictors* | *Estimates* | *95%CI* | *p-value* | *Estimates* | *95%CI* | *p-value* |
| (Intercept) | -0.23 | -0.97 – 0.52 | 0.55 | -1.48 | -2.16 – -0.79 | **<0.001** |
| Caffeine metabolic ratios^2^ | -0.30 | -0.40 – -0.20 | **<0.001** |  |  |  |
| Clozapine-to-norclozapine ratios |  |  |  | 0.14 | 0.04 – 0.23 | **0.004** |
| Age (10 years) | 0.08 | 0.03 – 0.13 | **0.001** | 0.08 | 0.03 – 0.14 | **0.004** |
| Sex (Female) | 0.13 | -0.05 – 0.31 | 0.17 | 0.10 | -0.10 – 0.30 | 0.335 |
| Body Mass Index | 0.03 | 0.01 – 0.05 | **0.003** | 0.04 | 0.02 – 0.06 | **0.001** |
| Smoking | -0.24 | -0.45 – -0.04 | **0.02** | -0.47 | -0.68 – -0.25 | **<0.001** |
| Time (hours)^3^ | -0.04 | -0.07 – -0.01 | **0.008** | -0.03 | -0.06 – 0.00 | 0.057 |
| Patients | 120 | | | 120 | | |
| Observations | 164 | | | 164 | | |
| Marginal R^2^ / Conditional R^2^ | 0.399 / 0.577 | | | 0.289 / 0.553 | | |
| Partial R^2^ for: |  |  |  |  |  |  |
| Caffeine metabolic ratios | 14.94% |  |  |  |  |  |
| Clozapine-to-norclozapine ratios |  |  |  | 4.18% |  |  |
| Age | 5.36% |  |  | 5.20% |  |  |
| Sex | 1.65% |  |  | 0.95% |  |  |
| Body Mass Index | 5.04% |  |  | 5.97% |  |  |
| Smoking | 2.83% |  |  | 9.32% |  |  |
| Time | 2.59% |  |  | 1.28% |  |  |

^1^ Log transformed to better approximate a normal distribution.

^2^ Calculated by dividing paraxanthine by caffeine plasma levels and multiplying the ratio by 1.07769 (Molecular mass ratio).

^3^ Time spent between last clozapine intake and blood sampling in hours.

Of note, regression coefficients for log-transformed variables represent proportional changes. Percent change = (e^^β^ - 1) × 100. For example: β = -0.30 → (e^-0.30 - 1) × 100 = -25.9% (a 25.9% decrease).

Abbreviations: CI=Confidence Interval; d=day; mg=milligram; ml=milliliter; ng=nanogram

Supplementary Table 10: Combined estimates, standard errors, and p values from 100 linear mixed-effects models fitted with imputed high-sensitivity C-reactive protein values

|  | **Clozapine [ng/mL] / dose** | | | **Olanzapine [ng/mL] / dose** | | |
| --- | --- | --- | --- | --- | --- | --- |
|  | **[mg/d] (N=164) ^1^** | | | **[mg/d] (N=222) ^1^** | | |
| *Predictors* | *Estimates* | *std.error* | *p-value* | *Estimates* | *std.error* | *p-value* |
| (Intercept) | -0.15 | 0.38 | 0.68 | 0.46 | 0.28 | 0.09 |
| Caffeine metabolic ratios ^2^ | -0.30 | 0.05 | **<0.001** | -0.04 | 0.03 | 0.22 |
| Age (10 years) | 0.01 | 0.00 | **0.008** | 0.00 | 0.00 | 0.10 |
| Sex (Female) | 0.09 | 0.09 | 0.31 | 0.26 | 0.09 | **0.003** |
| Body Mass Index | 0.03 | 0.01 | **0.006** | 0.02 | 0.01 | 0.058 |
| Smoking | -0.26 | 0.10 | **0.02** | -0.42 | 0.08 | **<0.001** |
| Time (hours)^3^ | -0.04 | 0.02 | **0.008** | -0.02 | 0.01 | **0.03** |
| High-sensitivity C-reactive protein quartile (ref < 0.876 mg/L) |  |  |  |  |  |  |
| [0.876,2.03 mg/L[ | -0.04 | 0.14 | 0.77 | 0.10 | 0.11 | 0.34 |
| [2.03,4.98 mg/L[ | 0.16 | 0.14 | 0.26 | 0.14 | 0.12 | 0.24 |
| [4.98,136 mg/L] | 0.20 | 0.14 | 0.15 | 0.11 | 0.12 | 0.35 |

^1^ Log transformed to better approximate a normal distribution.

^2^ Calculated by dividing paraxanthine by caffeine plasma levels and multiplying the ratio by 1.07769 (Molecular mass ratio).

^3^ Time spent between last clozapine or olanzapine intake and blood sampling in hours.

Of note, regression coefficients for log-transformed variables represent proportional changes. Percent change = (e^^β^ - 1) × 100. For example: β = -0.30 → (e^-0.30 - 1) × 100 = -25.9% (a 25.9% decrease).

Abbreviations: d=day; L=liter, mg=milligram; ml=milliliter; ng=nanogram; ref=reference; std=standard.

Supplementary Table 11: Multivariable model including clinical factors and GWAS-significant SNPs associated with caffeine metabolic ratios as covariates in Caucasians

|  | **Clozapine [ng/mL] / dose** | | | **Norclozapine [ng/mL] /** | | | **Clozapine [ng/mL] /** | | | **Olanzapine [ng/mL] / dose** | | |
| --- | --- | --- | --- | --- | --- | --- | --- | --- | --- | --- | --- | --- |
|  | **[mg/d]^1^** | | | **dose [mg/d] ^1^** | | | **Norclozapine [ng/mL] ^1^** | | | **[mg/d] ^1^** | | |
| *Predictors* | *Estimates* | *95%CI* | *p-value* | *Estimates* | *95%CI* | *p-value* | *Estimates* | *95%CI* | *p* | *Estimates* | *95%CI* | *p-value* |
| (Intercept) | -0.30 | -1.22 – 0.62 | 0.52 | -0.71 | -1.67 – 0.25 | 0.15 | 0.48 | -0.12 – 1.09 | 0.12 | 0.77 | 0.15 – 1.39 | **0.02** |
| Caffeine metabolic ratios ^2^ | -0.29 | -0.42 – -0.16 | **<0.001** | -0.18 | -0.31 – -0.06 | **0.003** | -0.10 | -0.19 – -0.01 | **0.04** | -0.11 | -0.19 – -0.03 | **0.007** |
| Age (10 years) | 0.09 | 0.02 – 0.16 | **0.01** | 0.07 | -0.01 – 0.14 | 0.07 | 0.02 | -0.03 – 0.06 | 0.49 | 0.06 | 0.01 – 0.12 | **0.02** |
| Sex (Female) | 0.15 | -0.10 – 0.40 | 0.23 | 0.08 | -0.19 – 0.36 | 0.55 | 0.08 | -0.09 – 0.24 | 0.35 | 0.25 | 0.07 – 0.44 | **0.008** |
| Body Mass Index | 0.02 | -0.00 – 0.05 | 0.10 | 0.03 | -0.00 – 0.05 | 0.06 | -0.01 | -0.02 – 0.01 | 0.50 | 0.01 | -0.01 – 0.03 | 0.23 |
| Smoking | -0.17 | -0.45 – 0.11 | 0.24 | -0.37 | -0.66 – -0.07 | **0.02** | 0.14 | -0.05 – 0.32 | 0.15 | -0.32 | -0.50 – -0.14 | **0.001** |
| Time (hours)^3^ | -0.02 | -0.06 – 0.01 | 0.22 | -0.03 | -0.07 – 0.00 | 0.06 | 0.01 | -0.02 – 0.03 | 0.46 | -0.03 | -0.05 – -0.00 | **0.03** |
| rs2472297 (TT or CT; ref: CC) | -0.26 | -0.52 – 0.01 | 0.06 | -0.10 | -0.39 – 0.19 | 0.51 | -0.16 | -0.33 – 0.02 | 0.08 | -0.05 | -0.24 – 0.15 | 0.65 |
| rs4410790 (TT or CT; ref: CC) | 0.05 | -0.21 – 0.31 | 0.69 | -0.04 | -0.32 – 0.24 | 0.77 | 0.11 | -0.05 – 0.28 | 0.18 | 0.07 | -0.13 – 0.26 | 0.50 |
| rs56113850 (TT or CT; ref: CC) | 0.12 | -0.14 – 0.38 | 0.36 | 0.11 | -0.17 – 0.40 | 0.43 | -0.02 | -0.19 – 0.15 | 0.78 | -0.07 | -0.27 – 0.14 | 0.51 |
| Patients | 56 | | | 56 | | | 56 | | | 98 | | |
| Observations | 95 | | | 95 | | | 95 | | | 143 | | |
| Marginal R^2^ / Conditional R^2^ | 0.398 / 0.617 | | | 0.314 / 0.715 | | | 0.131 / 0.341 | | | 0.304 / 0.655 | | |
| Partial R^2^ for: |  |  |  |  |  |  |  |  |  |  |  |  |
| Caffeine metabolic ratios | 5.67% |  |  | 0.00% |  |  | 4.86% |  |  | 1.29% |  |  |
| rs2472297 | 0.00% |  |  | 0.00% |  |  | 5.23% |  |  | 0.57% |  |  |
| rs4410790 | 0.00% |  |  | 0.00% |  |  | 4.86% |  |  | 1.23% |  |  |
| rs56113850 | 0.00% |  |  | 0.00% |  |  | 1.90% |  |  | 1.39% |  |  |

^1^ Log transformed to better approximate a normal distribution.

^2^ Calculated by dividing paraxanthine by caffeine plasma levels and multiplying the ratio by 1.07769 (Molecular mass ratio).

^3^ Time spent between last clozapine or olanzapine intake and blood sampling in hours.

Of note, regression coefficients for log-transformed variables represent proportional changes. Percent change = (e^^β^ - 1) × 100. For example: β = -0.29 → (e^-0.29 - 1) × 100 = -25.2% (a 25.2% decrease).

Abbreviations: CI=Confidence Interval; d=day; mg=milligram; ml=milliliter; ng=nanogram; ref=reference

Supplementary Table 12: Multivariable model including clinical factors and GWAS-significant SNPs associated with caffeine metabolic ratios as covariates in all ethnicities

|  | **Clozapine [ng/mL] / dose** | | | **Norclozapine [ng/mL] /** | | | **Clozapine [ng/mL] /** | | | **Olanzapine [ng/mL] / dose** | | |
| --- | --- | --- | --- | --- | --- | --- | --- | --- | --- | --- | --- | --- |
|  | **[mg/d] ^1^** | | | **dose [mg/d] ^1^** | | | **Norclozapine [ng/mL] ^1^** | | | **[mg/d] ^1^** | | |
| *Predictors* | *Estimates* | *95%CI* | *p-value* | *Estimates* | *95%CI* | *p-value* | *Estimates* | *95%CI* | *p-value* | *Estimates* | *95%CI* | *p-value* |
| (Intercept) | -0.22 | -1.12 – 0.68 | 0.63 | -0.71 | -1.68 – 0.25 | 0.14 | 0.53 | -0.04 – 1.10 | 0.07 | 0.37 | -0.23 – 0.97 | 0.22 |
| Caffeine metabolic ratios ^2^ | -0.29 | -0.41 – -0.16 | **<0.001** | -0.19 | -0.31 – -0.06 | **0.004** | -0.08 | -0.16 – -0.00 | **0.047** | -0.04 | -0.11 – 0.02 | 0.20 |
| Age (10 years) | 0.09 | 0.03 – 0.16 | **0.005** | 0.09 | 0.02 – 0.15 | **0.02** | 0.00 | -0.04 – 0.05 | 0.81 | 0.05 | 0.00 – 0.11 | **0.047** |
| Sex (Male) | 0.17 | -0.08 – 0.42 | 0.18 | 0.12 | -0.15 – 0.38 | 0.40 | 0.06 | -0.10 – 0.21 | 0.45 | 0.25 | 0.07 – 0.43 | **0.006** |
| Body Mass Index | 0.03 | -0.00 – 0.05 | 0.053 | 0.03 | 0.00 – 0.06 | **0.04** | -0.00 | -0.02 – 0.01 | 0.59 | 0.02 | 0.00 – 0.04 | **0.03** |
| Smoking | -0.18 | -0.45 – 0.09 | 0.20 | -0.33 | -0.63 – -0.04 | **0.03** | 0.12 | -0.05 – 0.29 | 0.17 | -0.38 | -0.55 – -0.22 | **<0.001** |
| Time (hours)^3^ | -0.03 | -0.07 – 0.00 | 0.08 | -0.04 | -0.08 – -0.00 | **0.04** | 0.01 | -0.02 – 0.03 | 0.56 | -0.02 | -0.04 – 0.01 | 0.15 |
| rs2472297 (TT or CT; ref: CC) | -0.24 | -0.51 – 0.03 | 0.08 | -0.06 | -0.36 – 0.23 | 0.66 | -0.17 | -0.34 – -0.00 | **0.048** | -0.02 | -0.23 – 0.19 | 0.87 |
| rs4410790 (TT or CT; ref: CC) | -0.02 | -0.26 – 0.23 | 0.89 | -0.14 | -0.40 – 0.13 | 0.32 | 0.13 | -0.02 – 0.29 | 0.09 | 0.09 | -0.10 – 0.27 | 0.35 |
| rs56113850 (TT or CT; ref: CC) | 0.07 | -0.19 – 0.33 | 0.60 | 0.07 | -0.22 – 0.35 | 0.64 | -0.02 | -0.18 – 0.14 | 0.78 | -0.12 | -0.32 – 0.09 | 0.26 |
| Patients | 62 | | | 62 | | | 62 | | | 139 | | |
| Observations | 103 | | | 103 | | | 103 | | | 210 | | |
| Marginal R^2^ / Conditional R^2^ | 0.431 / 0.595 | | | 0.354 / 0.625 | | | 0.119 / 0.315 | | | 0.248 / 0.749 | | |
| Partial R^2^ for: |  |  |  |  |  |  |  |  |  |  |  |  |
| Caffeine metabolic ratios | 13.00% |  |  | 5.47% |  |  | 3.27% |  |  | 0.60% |  |  |
| rs2472297 | 2.06% |  |  | 0.03% |  |  | 3.65% |  |  | 0.00% |  |  |
| rs4410790 | 0.00% |  |  | 0.16% |  |  | 3.27% |  |  | 0.55% |  |  |
| rs56113850 | 0.00% |  |  | 0.02% |  |  | 0.16% |  |  | 0.68% |  |  |

^1^ Log transformed to better approximate a normal distribution.

^2^ Calculated by dividing paraxanthine by caffeine plasma levels and multiplying the ratio by 1.07769 (Molecular mass ratio).

^3^ Time spent between last clozapine or olanzapine intake and blood sampling in hours.

Of note, regression coefficients for log-transformed variables represent proportional changes. Percent change = (e^^β^ - 1) × 100. For example: β = -0.29 → (e^-0.29 - 1) × 100 = -25.2% (a 25.2% decrease).

Abbreviations: CI=Confidence Interval; d=day; mg=milligram; ml=milliliter; ng=nanogram; ref=reference

# Supplementary figures

Supplementary figure 1: Factors associated with caffeine metabolic ratios in SKIPOGH considering genetics


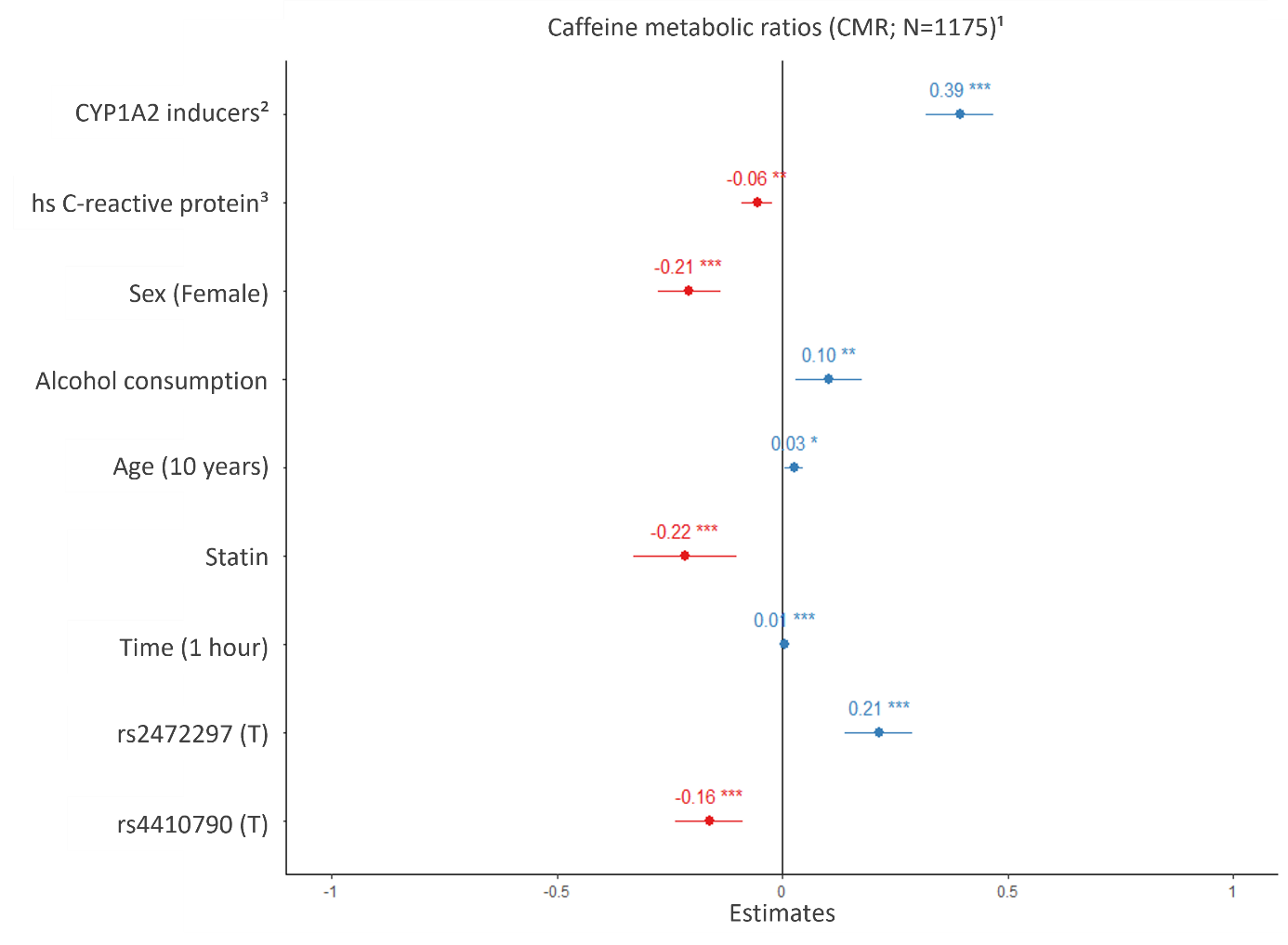


Number of observations varies due to missing values.

Covariates selected using backward procedure. Of note, due to the design of the SKIPOGH study (i.e., a multicenter study), the center effect was included in the analyses; however, as it failed to show statistically significant effect, it was excluded from the model.

*p<0.05, ** p<0.01, *** p<0.001.

^1^ Box-cox transformed to better approximate a normal distribution.

^2^ Including smoking

^3^ log transformed to insure normal distribution.

Abbreviations: hs=high-sensitivity.

It is noteworthy that rs2472297 and rs4410790 explained 3.3% and 2.5% of the variance of CMR, respectively, and accounting for the genetic variability the models improved slightly based on AIC criteria (AIC of model excluding genetic variability=2212, AIC of model considering genetic variability=1985).

Supplementary figure 2: Factors associated with caffeine metabolic ratios in CoLaus|PsyCoLaus considering genetics


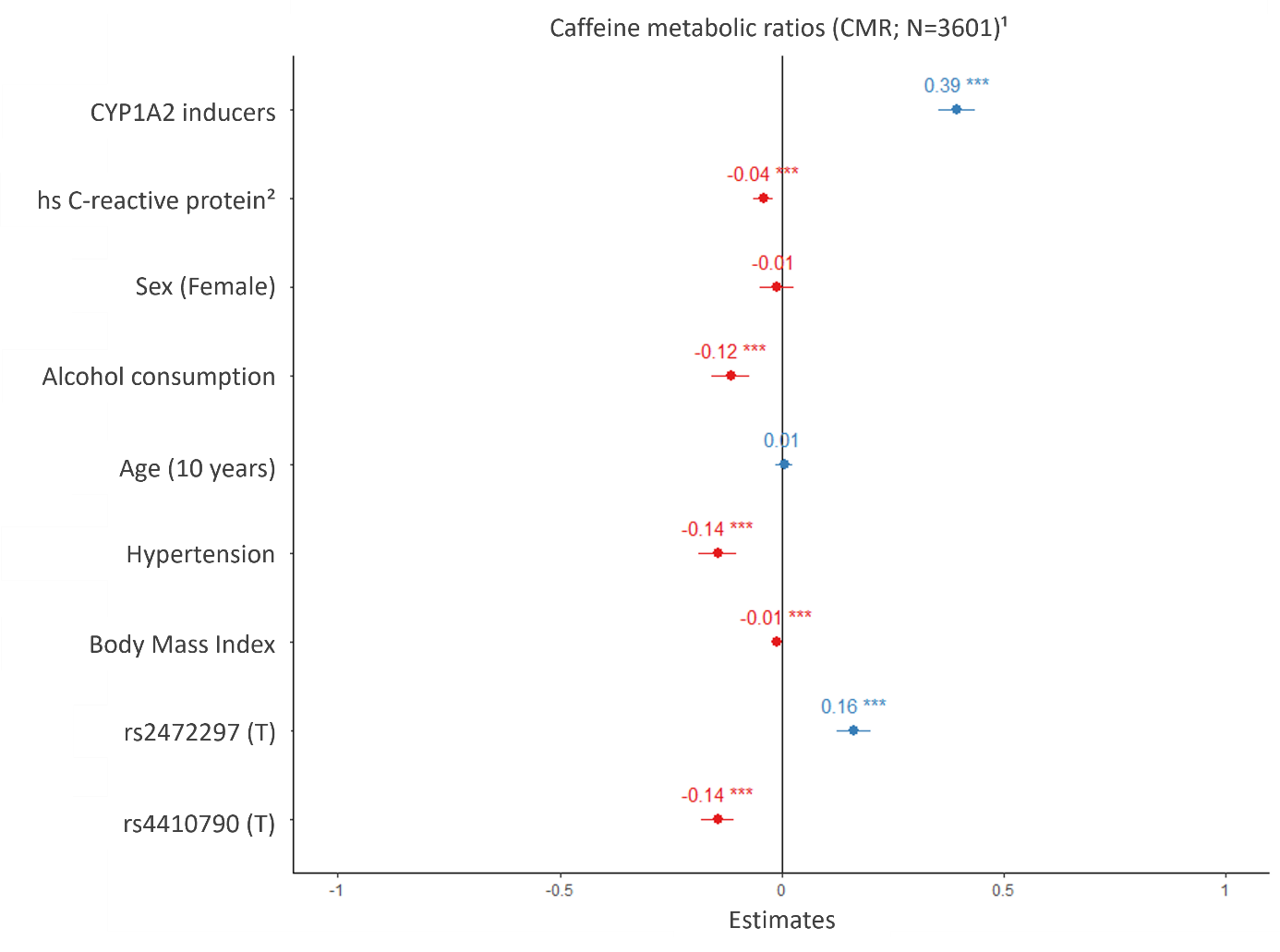


Number of observations varies due to missing values.

Covariates selected using backward procedure. CYP1A2 inducers included smoking.

*p<0.05, ** p<0.01, *** p<0.001.

^1^ Box-cox transformed to better approximate a normal distribution.

^2^ log transformed to insure normal distribution.

Abbreviations: hs=high-sensitivity.

It is noteworthy that rs2472297 and rs4410790 explained 1.9% and 1.6% of the variance of CMR, respectively, and accounting for the genetic variability the models improved slightly based on AIC criteria (AIC of model excluding genetic variability= 7651, AIC of model considering genetic variability= 5886).

Supplementary figure 3: Epigenome-Wide Association Study (EWAS) results for caffeine metabolic ratios (N=565)


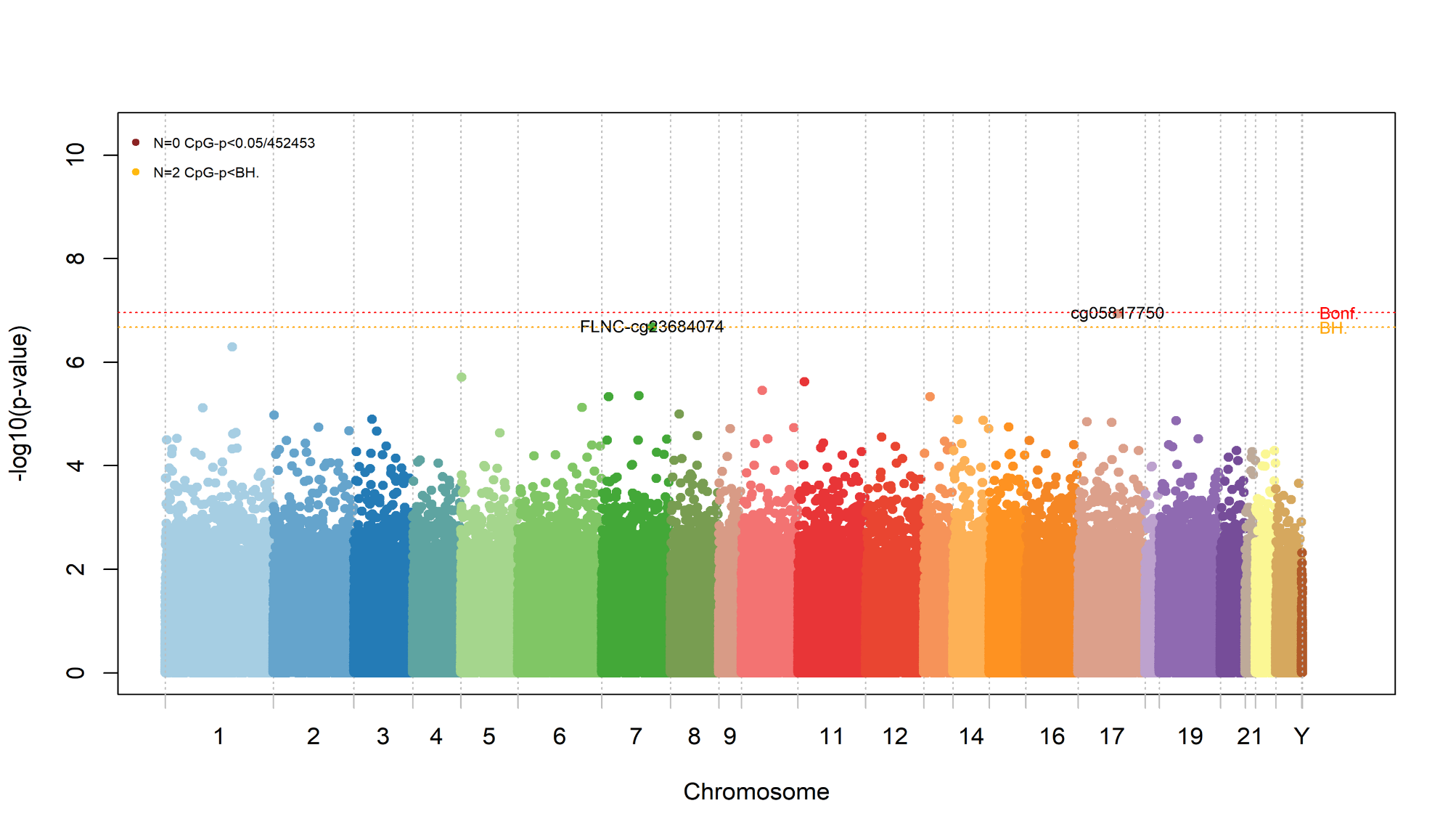


Linear regression model for the association between CpG markers and the caffeine metabolic ratios, adjusting for age, sex, recruitment center, seasonality of blood sampling, chip type, CPACOR principal components, body mass index, alcohol intake, the number of hours between blood sampling and last caffeine intake, CYP1A2 inducers, hypertension, statin intake, rs4410790, rs59251770, rs2472297, rs56113850 genotypes, and familial structure (random effect covariable).

Abbreviations: BH= Benjamini-Hochberg; Bonf= Bonferroni; CPACOR= control probe adjustment and reduction of global correlation.

Supplementary figure 4: Receiver operating characteristic curve analysis evaluating the performance of CMR to classify a twofold higher and a half lower value of the median dose-normalized plasma concentrations

**
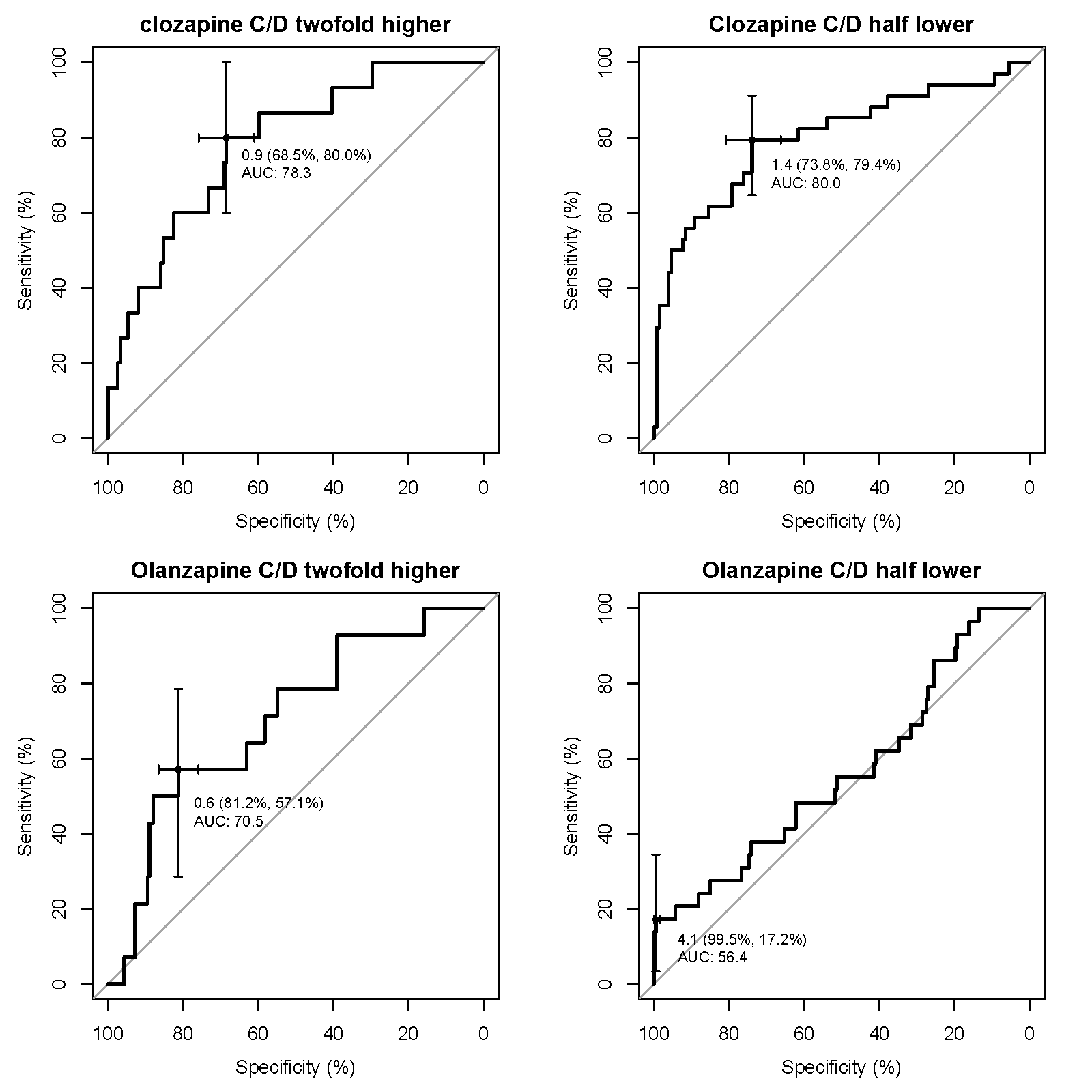
**
